# Mutations in myosin S2 alter cardiac myosin-binding protein-C interaction in hypertrophic cardiomyopathy in a phosphorylation-dependent manner

**DOI:** 10.1016/j.jbc.2021.100836

**Published:** 2021-05-27

**Authors:** Rohit R. Singh, James W. McNamara, Sakthivel Sadayappan

**Affiliations:** Division of Cardiovascular Health and Disease, Department of Internal Medicine, Heart, Lung and Vascular Institute, University of Cincinnati, Cincinnati, Ohio, USA

**Keywords:** Hypertrophic cardiomyopathy, myosin S2, *MYBPC3*, *MYH7*, phosphorylation, 4LP, four-parameter logistic curve, β-AR, β-adrenergic receptor, β-MyHC, β-myosin heavy chain, η, stoichiometry, AAA, phospho-ablated cMyBP-C or phospho-ablation, *B*_max_, maximal binding capacity, cMyBP-C, cardiac myosin-binding protein-C, DDD, phospho-mimetic cMyBP-C, DRX, disordered relaxed state, hC0-C2, human C0-C2, hC0-C2p, phosphorylation of hC0-C2, HCM, hypertrophic cardiomyopathy, hS2, human cardiac myosin S2, hS2^E924K^, human recombinant proximal S2 protein with E924K mutation, hS2^E930Δ^, human recombinant proximal S2 protein with E930Δ mutation, hS2^R870H^, human recombinant proximal S2 protein with R870H mutation, hS2^Wt^, human recombinant proximal S2 WT protein, ITC, isothermal titrating calorimetry, *K*_*d*_, dissociation constant, *k*_tr_, rate of force redevelopment, mC0-C2, mouse recombinant C0-C2 protein, *MYBPC3*, human cardiac myosin-binding protein-C gene, *MYH7*, β-myosin heavy-chain gene, S1, head of myosin, S2, subfragment 2 region, 126 amino acids of myosin, SPBA, solid-phase binding assay, SRX, super-relaxed

## Abstract

Hypertrophic cardiomyopathy (HCM) is an inherited cardiovascular disorder primarily caused by mutations in the β-myosin heavy-chain gene. The proximal subfragment 2 region (S2), 126 amino acids of myosin, binds with the C0-C2 region of cardiac myosin-binding protein-C to regulate cardiac muscle contractility in a manner dependent on PKA-mediated phosphorylation. However, it is unknown if HCM-associated mutations within S2 dysregulate actomyosin dynamics by disrupting its interaction with C0-C2, ultimately leading to HCM. Herein, we study three S2 mutations known to cause HCM: R870H, E924K, and E930Δ. First, experiments using recombinant proteins, solid-phase binding, and isothermal titrating calorimetry assays independently revealed that mutant S2 proteins displayed significantly reduced binding with C0-C2. In addition, CD revealed greater instability of the coiled-coil structure in mutant S2 proteins compared with S2^Wt^ proteins. Second, mutant S2 exhibited 5-fold greater affinity for PKA-treated C0-C2 proteins. Third, skinned papillary muscle fibers treated with mutant S2 proteins showed no change in the rate of force redevelopment as a measure of actin–myosin cross-bridge kinetics, whereas S2^Wt^ showed increased the rate of force redevelopment. In summary, S2 and C0-C2 interaction mediated by phosphorylation is altered by mutations in S2, which augment the speed and force of contraction observed in HCM. Modulating this interaction could be a potential strategy to treat HCM in the future.

Hypertrophic cardiomyopathy (HCM) is a global genetic heart disease affecting 1 in 500 people, including approximately 600,000 people in the United States and 14.25 million people worldwide. Importantly, cardiomyopathies, including HCM, are the most commonly identified causes of sudden cardiac death in young people and athletes (1, 2). Mutations in *MYH7* and *MYBPC3* sarcomeric genes encoding β-myosin heavy chain (β-MyHC) and cardiac myosin binding protein-C (cMyBP-C), respectively, account for 90% of HCM mutations (3, 4). Interestingly, these two proteins interact with each other to regulate cardiac muscle contraction, which suggests a potential common mechanism in the pathogenesis of HCM (5).

β-MyHC is a hexameric molecule comprised of two heavy chains and two pairs of light chains (Fig. 1*A*), and it is the motor protein responsible for cardiac contraction. β-MyHC can be divided into two parts, namely the N-terminal heavy meromyosin and the C-terminal light meromyosin (6). Heavy meromyosin can be further partitioned into globular S1 and a long coiled-coil subfragment 2 (7). Interestingly, the proximal subfragment 2 region (S2), 126 amino acids of myosin, is a hotspot for HCM-causing mutations (Table S1) (8, 9), although the mechanism by which these mutations result in disease remains unknown.

**Figure 1:**
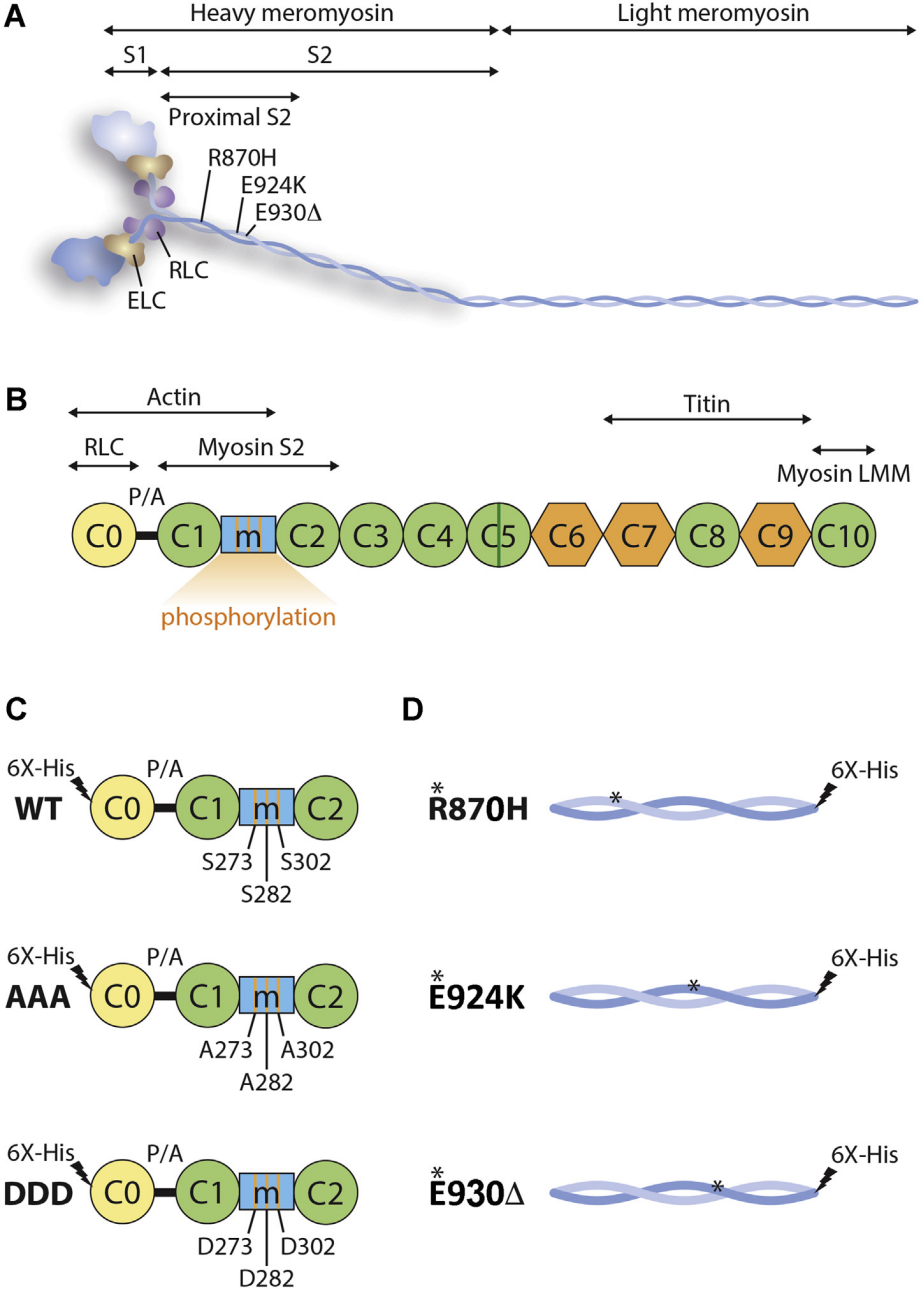
**Schematic diagram demonstrates the association between myosin and cMyBP-C.***A*, schematic diagram of myosin and cMyBP-C with their interaction domains. Myosin has been divided into N-terminal heavy meromyosin (HMM), which is further divided into S1 and S2 and the C-terminal light meromyosin (LMM). The long helical S2 is marked with proximal region of S2 (126 amino acids), three HCM-causing mutations, R870H, E924K, and E930Δ, and interacting regions with C0-C2 of cMyBP-C. The S1 head of myosin with essential light chain (ELC) and regulator light chain (RLC) proteins. *B*, in cMyBP-C, the diagram further displays cardiac-specific C0 domain, phosphorylation M domain, and a small inset in C5 domain (*green*-coded), proline-alanine (P/A)-rich region, interaction region with RLC, immunoglobulin domains from C1-C5, C8, and C10, and fibronectin domains, C6, C7, and C9. The phosphorylated M domain includes three major phosphorylation motifs (Ser-273, Ser-282, and Ser-302) that dynamically regulate the interaction with S2 region of myosin. *C*, schematic diagram of C0-C2 domains with WT, phospho-ablation (AAA), and phospho-mimetic (DDD), in which the phosphorylation sites Ser-273, Ser-282, and Ser-302 were mutated to either alanine or aspartic acid, respectively. *D*, proximal S2^Wt^, and three mutant hS2^R870H^, hS2^E924K^, and hS2^E930Δ^ proteins used in the present study. cMyBP-C, cardiac myosin-binding protein-C; hS2^E924K^, human recombinant proximal S2 protein with E924K mutation; hS2^E930Δ^, human recombinant proximal S2 protein with E930Δ mutation; hS2^R870H^, human recombinant proximal S2 protein with R870H mutation; S2, subfragment 2 region.

Stimulation of β-adrenergic receptors (β-ARs) activates PKA, which phosphorylates sarcomeric myofilament proteins, including cMyBP-C, to increase the speed of contraction (10). cMyBP-C is a 140-kDa sarcomeric thick filament protein (11) critical in the regulation of sarcomere structure and function in the heart (12). cMyBP-C connects both thick (myosin S2) and thin filament (actin and α-tropomyosin) proteins *via* its amino terminal (N’) region, including C0, C1, M, and C2 domains (C0-C2), and myosin light meromyosin and titin *via* its carboxyl terminal (C’) region (Fig. 1, *A* and *B*) (13, 14, 15). cMyBP-C possesses multiple M domain phosphorylation sites, including Ser-273, Ser-282, and Ser-302, which are absent in the two skeletal muscle isoforms. These sites are differentially targeted by PKA (10, 16), PKC (16, 17), Ca^2+^/calmodulin-dependent protein kinases II (10, 18), PKD (19), and ribosomal s6 kinase (20). PKA phosphorylates each of the above serines within the M domain (16, 21). These phosphorylation sites regulate the interaction between cMyBP-C and the S2 segment of β-MyHC (5, 22, 23, 24, 25). When the M domain is dephosphorylated, cMyBP-C strongly interacts with S2 (26), which decreases myosin cross-bridge duration and as a result depresses cardiac contractility (27, 28, 29, 30, 31). Upon cMyBP-C phosphorylation of the M domain serine sites, it loses its interaction with myosin S2, thereby allowing myosin heads to more readily interact with actin to accelerate the velocity and force of contraction (31, 32). Clearly, the interaction between S2 and C0-C2 is critical to thin and thick filament arrangements in the sarcomere and, hence, heart function (5, 24). Despite this importance, the exact molecular interactions and structure–function relationship between S2 and M domain remain unknown. Furthermore, to the best of our knowledge, no studies in the literature have reported the effect(s) of S2 mutations on their interaction with the C0-C2 region of cMyBP-C in a phosphorylation-dependent manner.

The recently published myosin mesa hypothesis suggests that the binding of C0-C2 to S2 allows myosin heads to adopt a structurally ordered state that limits their ability to interact with actin, known as the super-relaxed (SRX) state (33, 34). Another factor understood to regulate the SRX state is the interaction between myosin S1 heads and proximal myosin S2 (35, 36). However, in the absence of C0-C2 to S2 interaction, myosin heads transition out of the SRX state to the one termed as the disordered relaxed state (DRX) (26, 37, 38). Furthermore, HCM mutations affecting the S1 region of myosin resulted in a greater fraction of myosin S1 heads transitioning to the DRX state (39, 40, 41). Accordingly, the hypercontractile nature of HCM can be explained if myosin heads have transitioned into the DRX state, which would increase the total number of myosin heads available for contraction, leading to increased force of contractions (33, 34, 42, 43). Through this mechanism, the interaction of S2 with C0-C2 and myosin S1 plays a major role in determining the speed and force of contraction. Furthermore, we recently demonstrated that this SRX state is regulated by cMyBP-C phosphorylation through its binding to myosin S2 (33). Based on these data, we hypothesized that mutations in S2 affect its binding with C0-C2 and, hence, contractility. The present study tests this hypothesis by defining the *in vitro* binding of S2 and C0-C2 of cMyBP-C in the context of S2 mutations and C0-C2 phosphorylation, respectively (25, 44). We studied the biochemical and functional properties of three HCM-causing mutations in the S2: R870H (45), E924K (46), and E930Δ (47). Although these mutations significantly reduced the interaction of S2 and C0-C2 domain of cMyBP-C, we found that they unexpectedly increased their affinity for the C0-C2 domain of cMyBP-C upon PKA phosphorylation, compared with WT S2 controls. Overall, our studies demonstrate that myosin S2 and C0-C2 interactions, which are mediated by phosphorylation, play a major role in regulating contractility, consistent with the mutation roles as causative for HCM.

## Results

To define whether HCM-associated mutations within the proximal 126 residues (838–963) of human cardiac myosin S2 (hS2) affect its interaction with human C0-C2 (hC0-C2) region of cMyBP-C (Fig. S1), we used three different HCM-causing mutations, R870H (human recombinant proximal S2 protein with R870H mutation [hS2^R870H^]), E924K (human recombinant proximal S2 protein with E924K mutation [hS2^E924K^]), and E930Δ (human recombinant proximal S2 protein with E930Δ mutation [hS2^E930Δ^]) (46, 47), compared with WT hS2 (human recombinant proximal S2 WT protein [hS2^Wt^]) controls. The amino acid sequence of the S2 region of human β-MyHC and mouse α-MyHC is highly conserved (Fig. S2). The interaction partner mouse recombinant C0-C2 protein (mC0-C2) domains of cMyBP-C with phospho-ablation (AAA) and phospho-mimetic mutations were used in the study to define their affinities to hS2 (Fig. 1, *C* and *D*). Recombinant proteins with a 6X His-tag were expressed in BL21 bacterial cells and purified through His-tag affinity purification (Fig. S3). The three phosphorylation sites in the M domain of cMyBP-C are highly phosphorylated under basal conditions, as required for regular cardiac function (12, 18, 32). The interaction of the S2 region with cMyBP-C is modulated by phosphorylation and dephosphorylation of cMyBP-C within the M domain to regulate increased or decreased speed of contraction, respectively (12, 18, 32). Based on these studies, we hypothesized that mutations in the S2 region affect the S2–C0-C2 interaction differentially either in presence or absence of C0-C2 phosphorylation. Therefore, the following independent experiments were performed in the presence and absence of C0-C2 phosphorylation to determine if these post-translational modifications affect S2 binding in the presence and absence of the indicated mutations. *In vitro* phosphorylation of hC0-C2 (hC0-C2p) was achieved by treating the hC0-C2 proteins with PKA, and the C0-C2 phosphorylation level was confirmed by Western blot analysis using phospho-site-specific antibodies (Fig. S4).

### WT and mutant S2 peptides display divergent binding affinity for phosphorylated C0-C2 proteins

Solid-phase binding assay (SPBA) experiments were first performed to define whether the S2 mutations affect their maximal binding capacity (*B*_*max*_), which indicates the level of available binding sites (molar ratios). The rate of equilibrium dissociation constant (*K*_*d*_) is a measure of binding affinity. In the present study, binding properties (*B*_*max*_ and *K*_*d*_) of the mutant S2 proteins with hC0-C2 protein in the presence and absence of phosphorylation were determined, compared with hS2^Wt^ proteins. For this experiment, we first generated antibody against hS2 peptide (838-PLLKSAEREKEMASMKEE-855 codons) and validated its specificity by Western blot (Fig. S6), as well as upper and lower detection limits of anti-S2 antibody and anti-C0 domain-specific cMyBP-C antibody (21) by ELISA (Fig. S7, Table S2).

**Figure 2:**
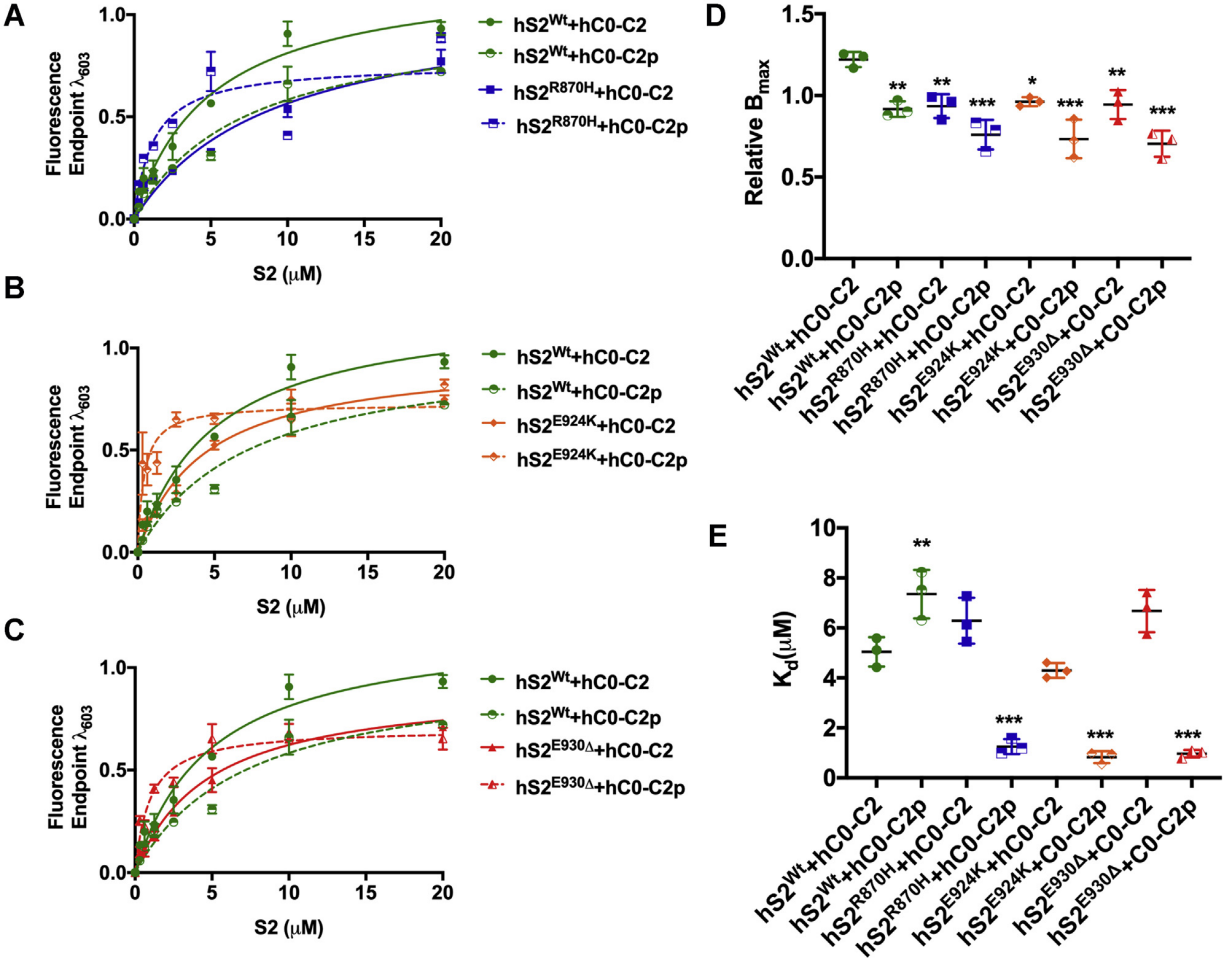
**Mutant hS2 proteins display increased affinity by decreasing *K***_***d***_**to phosphorylated hC0-C2 protein by SPBA.** The binding curve between hS2^Wt^ and hC0-C2 (*solid green*), hS2^Wt^ and hC0-C2p (*dashed green*), (*A*) hS2^R870H^ and hC0-C2 (*solid blue*), hS2^R870H^ and hC0-C2p (*dashed blue*), (*B*) hS2^E924K^ and hC0-C2 (*solid orange*), hS2^E924K^ and hC0-C2p (*dashed orange*), (*C*) hS2^E930Δ^and hC0-C2 (*solid red*), and hS2^E930Δ^and hC0-C2p (*dashed red*). *D*, relative maximal binding capacity (*B*_max_) was determined for each listed combination and compared against hS2^Wt^ and C0-C2 proteins. *E*, binding affinity or dissociation constant, *K*_*d*_, compared against hS2^Wt^ and hC0-C2 proteins. Statistical analyses were performed by one-way ANOVA with Tukey’s multiple comparison test with single pooled variance. n = 3 with triplicates for each concentration. Data are expressed as the mean ± SEM. See Table 1 for analysis of main factors and interactions. ∗*p* < 0.05, ∗∗*p* < 0.01, and ∗∗∗*p* < 0.001 *versus* hS2^Wt^/hC0-C2 controls. hS2^E924K^, human recombinant proximal S2 protein with E924K mutation; hS2^E930Δ^, human recombinant proximal S2 protein with E930Δ mutation; hS2^R870H^, human recombinant proximal S2 protein with R870H mutation; hS2^Wt^, human recombinant proximal S2 WT protein; S2, subfragment 2 region; SPBA, solid-phase binding assay.

Concentration-dependent binding of hS2^Wt^ and mutant S2 proteins at 20 μM was established using various concentrations of hC0-C2 (0, 1, 3, 5, 10, and 20 μM) (Fig. S8). From this initial experiment, we determined that *B*_max_ of each mutant hS2 (hS2^R870H^, hS2^E924K^, and hS2^E930Δ^) with dephosphorylated hC0-C2 was significantly reduced compared with the values obtained using hS2^Wt^ (Fig. 2, *A*–*C* and Table 1). Compared with hS2^Wt^ (*B*_max_ = 1.22 ± 0.06), the *B*_max_ of mutant hS2 protein was significantly decreased by approximately 24% across all mutants: hS2^R870H^ (0.93 ± 0.04, ∗∗*p* < 0.01), hS2^E924K^ (0.96 ± 0.02, ∗*p* < 0.05), and hS2^E930Δ^ (0.94 ± 0.05, ∗∗*p* < 0.01) (Fig. 2*D* and Table 1). Based on *B*_*max*_ values, the stoichiometry (η) for hC0-C2 to hS2 mutants compared with hS2^WT^ increased to 1.24 for hS2^R870H^, 1.21 for hS2^E924K^, and 1.23 for hS2^E930Δ^, thus stating more hS2 mutant proteins were required to saturate the hC0-C2 than hS2^WT^. When hS2^Wt^ protein was allowed to interact with hC0-C2p, *B*_max_ was significantly reduced (0.92 ± 0.03, ∗∗*p* < 0.01) compared with hC0-C2 control (1.22 ± 0.06) (Fig. 2, *A*–*D* and Table 1), confirming that S2^Wt^ binding is reduced when the M-domain in C0-C2 is phosphorylated. The η, based on *B*_*max*_, for hC0-C2p to hS2^Wt^ was increased to 1.26 compared with that of hC0-C2 to hS2^WT^. The *B*_max_ to hC0-C2p using the mutant S2 proteins was further reduced with no significant difference: hS2^R870H^ (0.76 ± 0.05), hS2^E924K^ (0.73 ± 0.07), and hS2^E930Δ^ (0.70 ± 0.04), compared with that of hS2^Wt^ to hC0-C2 protein (0.92 ± 0.03). With the *B*_*max*_, the η for hC0-C2p to hS2 mutants increased to 1.17 for hS2^R870H^, 1.21 for hS2^E924K^, and 1.24 for hS2^E930Δ^. Overall, the *B*_max_ was lowered by an average of 18% in mutant hS2 proteins, compared with hS2^Wt^ with hC0-C2p proteins. These data suggest that the binding capacity of the three mutant hS2 proteins to hC0-C2 is strongly reduced, both at baseline and when the M domain is phosphorylated.

**Table 1:** Maximal binding capacity (*B*_max_) and dissociation constants (*K*_*d*_) for binding of prey proteins, hS2, to bait proteins, hC0-C2, with or without PKA in the SPBA experiments

Prey	Bait	n	*B*_max_ (mol/mol: S2/C0-C2)	*K*_*d*_ (μM)
hS2^Wt^	hC0-C2	3	1.22 ± 0.02	5.04 ± 0.33
hS2^R870H^		3	0.93 ± 0.04∗∗	6.29 ± 0.53
hS2^E924K^		3	0.96 ± 0.02∗	4.30 ± 0.17
hS2^E930Δ^		3	0.94 ± 0.05∗∗	6.67 ± 0.48
hS2^Wt^	hC0-C2p	3	0.92±0.03∗∗	7.35 ± 0.56∗∗
hS2^R870H^		3	0.76±0.05∗∗∗	1.26 ± 0.17∗∗∗^, §§§, ¶¶¶^
hS2^E924K^		3	0.73±0.07∗∗∗	0.83 ± 0.13∗∗∗^, §§§, ¶¶¶^
hS2^E930Δ^		3	0.70±0.04∗∗∗	0.97 ± 0.08∗∗∗^, §, ¶¶¶^

Data are expressed as the mean ± SEM for the observed values. Statistical analyses were performed in all groups using ordinary one-way ANOVA, followed by Tukey’s multiple comparison test with single pooled variance. See Table 7 for analysis of main factors and interactions. ‘n’ is the times binding experiments were performed independently in replicates of three.

∗*p* < 0.05, ∗∗*p* < 0.01, ∗∗∗*p* < 0.001 *versus* hS2Wt /hC0-C2.

^§^*p* < 0.05, ^§§^*p* < 0.01, ^§§§^*p* < 0.001 *versus* hS2Wt/hC0-C2p.

^¶^*p* < 0.05, ^¶¶^*p* < 0.01, ^¶¶¶^*p* < 0.001 *versus* respective mutant/hC0-C2.

*K*_*d*_ values of hS2^Wt^ (5.04 ± 0.33) to hC0-C2 protein were measured and compared with the mutant hS2 proteins (Fig. 2*E* and Table 1). Interestingly, the *K*_*d*_ values of the three mutant hS2 proteins were similar to those of hS2^Wt^ proteins, suggesting the absence of any difference in the strength of binding of all hS2 proteins with hC0-C2 proteins (Fig. 2*E* and Table 1). Next, the impact of C0-C2 phosphorylation on its binding affinity to hS2^Wt^ was assessed. As expected, the *K*_*d*_ values for hS2^Wt^ to hC0-C2p were significantly increased (7.35 ± 0.56 μM *versus* 5.04 ± 0.33, ∗∗*p* < 0.01) compared with the hC0-C2 dephosphorylated control (Fig. 2*E* and Table 1). Strikingly, all three mutant hS2 proteins displayed significantly decreased *K*_*d*_ values: hS2^R870H^ (1.26 ± 0.16 μM, 6-fold decrease, ∗∗∗*p* < 0.001), hS2^E924K^ (0.83 ± 0.05 μM, 9-fold decrease, ∗∗∗*p* < 0.001), and hS2^E930Δ^ (0.97± 0.05 μM, ∗∗∗*p* < 0.001), compared with that of hS2^Wt^ to hC0-C2p (7.35 ± 0.56 μM) (Fig. 2*E* and Table 1). Therefore, when C0-C2 is phosphorylated, the mutant S2 proteins show an increased affinity with hC0-C2p. In summary, we observed no difference in the binding affinity of hS2^Wt^ and mutant hS2 to hC0-C2. In contrast, hS2^Wt^ exhibited weaker binding affinity to hC0-C2p, whereas the mutant hS2 showed greater binding affinity with hC0-C2p.

**Figure 3:**
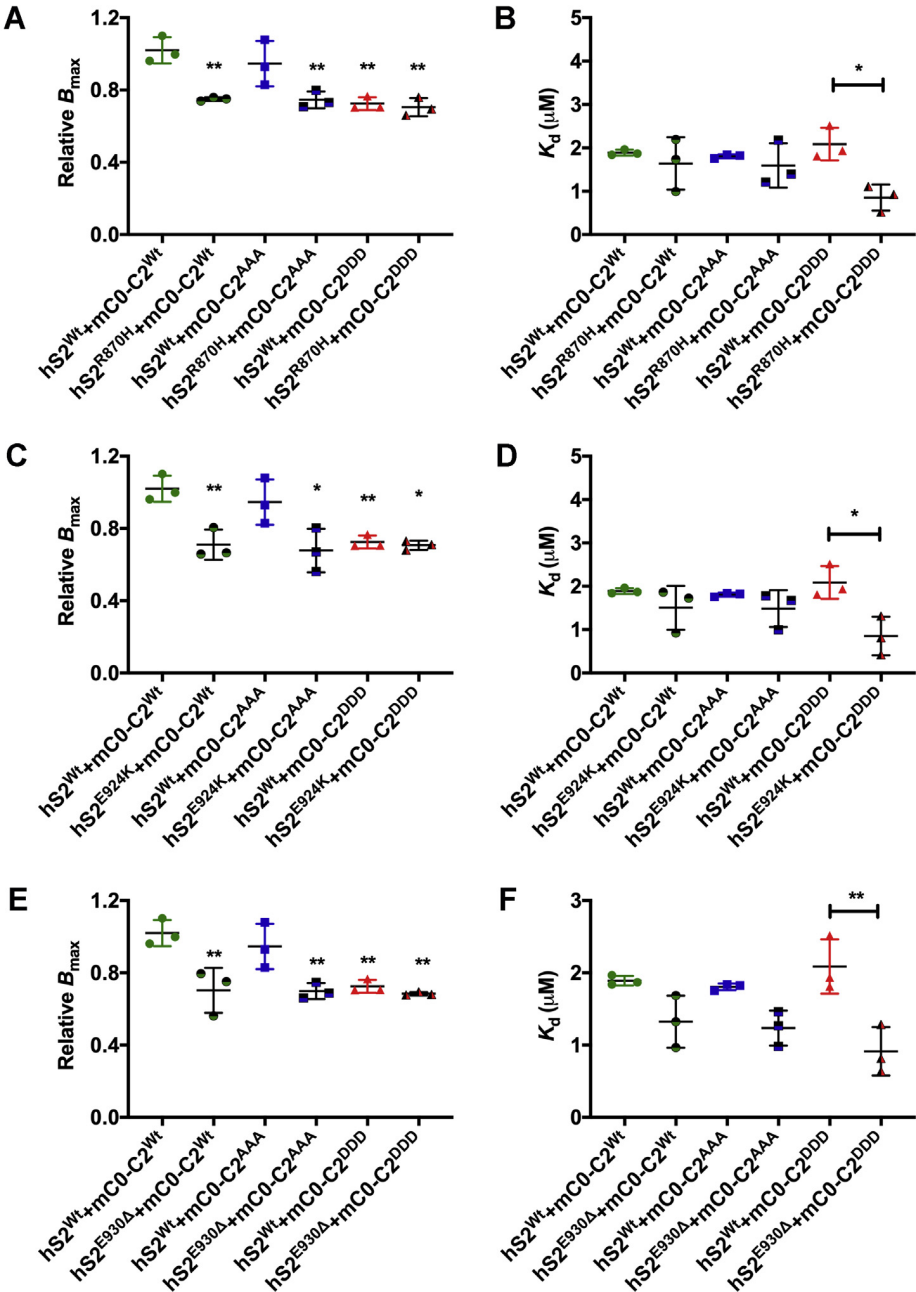
**Increased affinity of mutant hS2 proteins to mC0-C2**^**DDD**^**protein by SPBA.***A*, relative *B*_max_ and *B*, relative *K*_*d*_ for mC0-C2^Wt^ (*green*), mC0-C2^AAA^ (*blue*), and mC0-C2^DDD^ (*red*) treated with hS2^Wt^ (*solid line*) and S2^R870H^ (*dashed line*). *C*, relative *B*_max_ and *D*, relative *K*_*d*_ for mC0-C2^Wt^ (*green*), mC0-C2^AAA^ (*blue*), and mC0-C2^DDD^ (*red*) treated with hS2^Wt^ (*solid line*) and hS2^E924K^ (*dashed line*). *E*, relative *B*_max_ and *F*, relative *K*_*d*_ for mC0-C2^Wt^ (*green*), mC0-C2^AAA^ (*blue*), and mC0-C2^DDD^ (*red*) treated with hS2^Wt^ (*solid line*) and hS2^E930Δ^ (*dashed line*). Data were fit to Michaelis–Menten binding fit, and one-way ANOVA with Tukey’s pooled multiple variance test were used to calculate the significance between the *K*_*d*_ values. n = 3 with replicates of three for each n value. Data are expressed as the mean ± SEM. See Table 2 for analysis of main factors and interactions. ∗*p* < 0.05, ∗∗*p* < 0.01 *versus* hS2^Wt^ to mC0-C2^Wt^ control. AAA, phospho-ablated cMyBP-C or phospho-ablation; DDD, phospho-mimetic cMyBP-C; hS2^E930Δ^, human recombinant proximal S2 protein with E930Δ mutation; hS2^Wt^, human recombinant proximal S2 WT protein; *K*_*d*_, dissociation constant; mC0-C2, mouse recombinant C0-C2 protein; SPBA, solid-phase binding assay.

Previously, cardiac-specific transgenic mouse models expressing phospho-mimetic (25) or AAA (32) cMyBP-C were used to demonstrate the *in vivo* role(s) of cMyBP-C phosphorylation (12). Therefore, to independently verify whether the increased affinity of mutant S2 proteins selectively resulted from phosphorylation of the C0-C2 region, we used recombinant mouse C0-C2 proteins in which the phosphorylation sites were mutated to either alanine (mC0-C2^AAA^) or aspartic acids (mC0-C2^DDD^) to mimic dephosphorylated and phosphorylated C0-C2, respectively, compared with WT control (mC0-C2^Wt^). Consistent with above results, the *B*_max_ was significantly reduced for all hS2 mutants binding to mC0-C2^AAA^ and mC0-C2^DDD^, compared with hS2^Wt^ (Fig. 3, *A*, *C* and *E*, Fig. S9 and Table 2). Also, in agreement with the previous results, mutant S2 proteins significantly decreased *K*_*d*_ with only phospho-mimetic mC0-C2^DDD^ protein, as follows: hS2^R870H^ (0.79 ± 0.19 μM, †*p* = 0.051, §*p* = 0.08), hS2^E924K^ (0.74 ± 0.21, †∗*p* < 0.05, §*p* = 0.06), and hS2^E930Δ^ (0.88 ± 0.10 μM, †∗∗*p* < 0.01, §∗*p* < 0.05), compared with mC0-C2^Wt^ (†1.89 ± 0.03 μM) and mC0-C2^AAA^ (§2.02 ± 0.56 μM) (Fig. 3, Fig. S9 and Table 2). These data validate that the binding properties of both mouse and hC0-C2 proteins were similar with hS2 proteins despite the small differences in the amino acids among the species (Figs. S1 and S2). Taken together, we conclude that although mutant S2 proteins have reduced binding capacity to dephosphorylated C0-C2, they bind more tightly to phosphorylated C0-C2, than the S2^Wt^.

**Table 2:** Maximal binding capacity (*B*_max_) and dissociation constants (*K*_*d*_) for binding of prey proteins, hS2, to bait proteins, hC0-C2, with phospho-ablation and phospho-mimetic in the SPBA experiments

Prey	Bait	n	*B*_max_ (mol/mol S2/C0-C2)	*K*_*d*_ (μM)
hS2^Wt^	mC0-C2	3	1.02 ± 0.04	1.89 ± 0.03
hS2^R870H^		3	0.75 ± 0.01∗∗	1.64 ± 0.34
hS2^E924K^		3	0.71 ± 0.05∗∗	1.51 ± 0.29
hS2^E930Δ^		3	0.70 ± 0.01∗∗	1.32 ± 0.21
hS2^Wt^	mC0-C2^AAA^	3	0.95 ± 0.04	1.81 ± 0.32
hS2^R870H^		3	0.74 ± 0.03∗∗^,§^	1.51± 0.22
hS2^E924K^		3	0.67 ± 0.04∗∗^,§^	1.48 ± 0.24
hS2^E930Δ^		3	0.70 ± 0.02∗∗^,§^	1.22 ± 0.13
hS2^Wt^	mC0-C2^DDD^	3	0.70 ± 0.04∗∗^,§§^	2.02 ± 0.56
hS2^R870H^		3	0.70 ± 0.04∗∗^,§^	0.79 ± 0.19^¶^
hS2^E924K^		3	0.73 ± 0.11∗∗^,§^	0.74 ± 0.21∗^,¶^
hS2^E930Δ^		3	0.68 ± 0.02∗∗^,§^	0.88 ± 0.10∗∗^,§,¶¶^

Statistical significance was calculated by performing ordinary one-way ANOVA with Tukey’s multiple comparison test with single pooled variance. ‘n’ is the times binding experiments were performed independently in replicates of three. ± values represent the SEM for the measurements. See Table 7 for analysis of main factors and interactions.

∗*p* < 0.05, ∗∗*p* < 0.01, ∗∗∗*p* < 0.001 *versus* hS2^Wt^/mC0-C2.

^§^*p* < 0.05, ^§§^*p* < 0.01, ^§§§^*p* < 0.001 *versus* hS2^Wt^/mC0-C2^AAA^.

^¶^*p* < 0.05, ^¶¶^*p* < 0.01, ^¶¶¶^*p* < 0.001 *versus* hS2^Wt^/mC0-C2^DDD^.

### Isothermal titrating calorimetry assay independently validated the SPBA results

**Figure 4:**
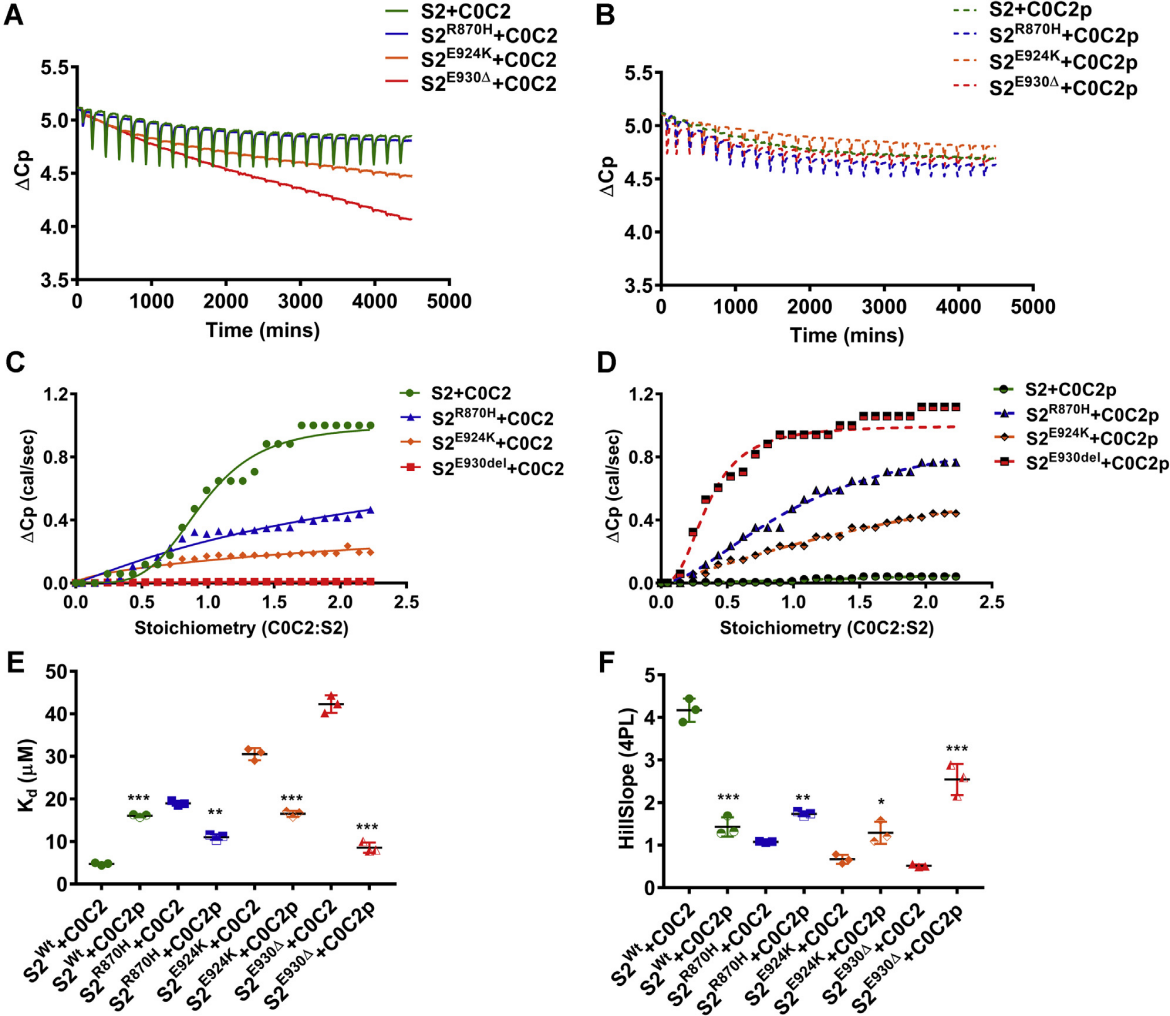
**Mutant hS2 displays a higher rate of heat change upon binding to phosphorylated hC0-C2 protein by ITC assay.***A*, representative traces for change in heat observed upon titrating hS2^Wt^ (*green*), hS2^R870H^ (*blue*), hS2^E924K^ (*orange*), and E930Δ (*red*) proteins to hC0-C2 proteins. *B*, representative traces for change in heat observed upon titrating hS2^Wt^ (*green dashed*), hS2^R870H^ (*blue dashed*), hS2^E924K^ (*orange dashed*), and hS2^E930Δ^ (*red dashed*) proteins to hC0-C2p proteins. *C*, sigmoidal curve for titration of hS2^Wt^ to hC0-C2 yields the dissociation constant (1/slope) and stoichiometry of the reaction. Titration of hS2^Wt^ (*green*), hS2^R870H^ (*blue*), hS2^E924K^ (*orange*), and hS2^E930Δ^ (*red*) proteins against dephosphorylated hC0-C2 proteins. *D*, sigmoidal curve for titration of hS2^Wt^ (*green dashed*), hS2^R870H^ (*blue dashed*), hS2^E924K^ (*orange dashed*), and hS2^E930Δ^ (*red dashed*) proteins to phosphorylated hC0-C2 protein. *E*, change in affinity for hS2^Wt^ to hC0-C2 (*solid*) and hC0-C2p (*half solid*) proteins. *F*, change in slope for hS2^Wt^ proteins to hC0-C2 (*solid*) and hC0-C2p (*half solids*) protein calculated by four-parameter logistic curve. Statistical analyses were performed by one-way ANOVA with Tukey’s multiple comparison test with single pooled variance. n = 3, where each n value was performed for hC0-C2 and hC0-C2p to minimize variance. Data are expressed as the mean ± SEM. See Table 3 for analysis of main factors and interactions. ∗*p* < 0.05, ∗∗*p* < 0.01, and ∗∗∗*p* < 0.001 *versus* hS2^Wt^ to hC0-C2 control. hS2, human cardiac myosin S2; hS2^E924K^, human recombinant proximal S2 protein with E924K mutation; hS2^E930Δ^, human recombinant proximal S2 protein with E930Δ mutation; hS2^R870H^, human recombinant proximal S2 protein with R870H mutation; hS2^Wt^, human recombinant proximal S2 WT protein; ITC, isothermal titrating calorimetry; S2, subfragment 2 region.

To determine the preferential binding of hS2 to hC0-C2 in the presence of S2 mutations and the impact of hC0-C2 phosphorylation, we next performed an isothermal titrating calorimetry (ITC) assay. For this experiment, 350 μM of either WT or mutant hS2 was titrated to 20 μM of either basal or phosphorylated hC0-C2 (Fig. 4*A*). Results showed little or no change in heat capacity (ΔCp) in *K*_*d*_ per second between hS2^Wt^ titrated to hC0-C2p compared with hS2^Wt^ and hC0-C2 (Fig. 4*B*). The heat exchange data from ITC experiments revealed *K*_*d*_ of hS2^Wt^ to hC0-C2 at 4.71 μM and η of 1.08 at 20 °C, values consistent with previously published ITC data (5) (Fig. 4*C* and Table 3). In contrast, hardly any ΔCp differences were observed when S2^Wt^ was titrated to hC0-C2p (Fig. 4*D* and Table 3). Therefore, data could not be fit to yield a measurable *K*_*d*_, and η was found to be 22.58, which was outside the range for the concentration of proteins used, thus confirming highly selective binding of the S2 region to dephosphorylated N-terminal cMyBP-C.

**Table 3:** Comparison of slope of heat change, affinity (*B*_max_), and stoichiometry between various hS2/hC0-C2 interactions in the ITC experiment

Prey	Bait	n	Slope	*K*_*d*_ (μM)	Stoichiometry (η)
hS2^Wt^	hC0-C2	3	4.18 ± 0.2	4.71 ± 0.17	1.08 ± 0.01
hS2^R870H^		3	1.08 ± 0.08∗∗∗	18.99 ± 0.32∗∗∗	2.53 ± 0.14
hS2^E924K^		3	0.65 ± 0.06∗∗∗	30.52 ± 0.81∗∗∗	15.88 ± 3.87^a^
hS2^E930Δ^		3	0.5 ± 0.05∗∗∗	42.27 ± 1.18∗∗∗	22,169^a^
hS2^Wt^	hC0-C2p	3	1.28 ± 0.14∗∗∗	16.06 ± 0.18∗∗∗	22.33 ± 6.46^a^
hS2^R870H^		3	1.74 ± 0.05∗∗∗^,¶^	11.03 ± 0.37∗∗∗^,§§§,¶¶^	1.07 ± 0.01
hS2^E924K^		3	1.20 ± 0.03∗∗∗	16.51 ± 0.42∗∗∗^,¶¶¶^	2.57 ± 0.05
hS2^E930Δ^		3	2.58 ± 0.3∗∗∗^,§§§,¶¶¶^	8.57 ± 0.69∗∗^,§§§,¶¶¶^	0.35 ± 0.02

Statistical significance was calculated by performing ordinary one-way ANOVA with Tukey’s multiple comparison test with single pooled variance. Affinity value is calculated by the reciprocal of the slope to yield concentration of hS2 bound to hC0-C2 protein. ‘n’ is equal to the number of independent experiments performed. ± values are the SEM for measurements performed. See Table 7 for analysis of main factors and interactions.

∗*p* < 0.05, ∗∗*p* < 0.01, ∗∗∗*p* < 0.001 *versus* hS2Wt /hC0-C2.

^§^*p* < 0.05, ^§§^*p* < 0.01, ^§§§^*p* < 0.001 *versus* hS2Wt/hC0-C2p.

^¶^*p* < 0.05, ^¶¶^*p* < 0.01, ^¶¶¶^*p* < 0.001 *versus* respective mutant hS2/hC0-C2.

a Indicates the values outside of the experimental values.

Having defined the ITC values for hS2^WT^, we next validated results from our SBPA. We performed ITC for each hS2 HCM variant at 350 μM and titrated against 20 μM of hC0-C2 (+/− PKA phosphorylation). While the ΔCp for hS2^R870H^ was higher than that produced by hS2^E924K^ or hS2^E930Δ^, all hS2 mutants exhibited ΔCp values significantly lower than hS2^Wt^, indicating a reduced interaction (Fig. 4*A*). This was evident by the reduced Hill slope for each mutant (hS2^R870H^ 1.08, hS2^E924K^ 0.65, and hS2^E930Δ^ 0.5) compared with hS2^Wt^ (4.18, all mutants *p* < 0.001). Similarly, the *K*_*d*_ was drastically higher for each hS2 mutant than the WT (hS2^Wt^ = 4.71 ± 0.17, hS2^R870H^ = 18.99 ± 0.32, hS2^E924K^ = 30.52 ± 0.81, and hS2^E930Δ^ = 42.27 ± 1.18) (Fig. 4*E* and Table 3). The η for HCM mutants was higher than the concentration of proteins in the assay (hS2^R870H^ 2.53, hS2^E924K^ 15.88, and hS2^E930Δ^ 22,169), which further demonstrates that the binding between mutant hS2 and hC0-C2 proteins was reduced (Table 3). Of note, S2^R870H^ was the only mutant hS2 with a measurable η and a higher Hill slope, demonstrating that although its binding affinity to hC0-C2 was less than hS2^Wt^, it was not reduced to the extent exhibited by hS2^E924K^ and hS2^E930Δ^ proteins.

We then determined whether PKA treatment of hC0-C2 affected its interactions with mutant hS2 proteins. The ΔCp per second was higher across mutant hS2 proteins for hC0-C2p protein than their binding with hC0-C2 protein, as described above (Fig. 4*B*). When the values for ΔCp were fitted to the four-parameter variable slope, all mutant hS2 proteins had higher slope values when titrated to phosphorylated hC0-C2p than hC0-C2 titration (Table 3). Furthermore, the slope values expressed as a four-parameter logistic curve (4PL), were significantly increased by 1.6 4PL (∗∗*p* < 0.001), 1.9 4PL (∗∗*p* < 0.001), and 5.2 4PL (∗∗*p* < 0.001) times for hS2^R870H^, hS2^E924K^, and hS2^E930Δ^, respectively, when titrated to hC0-C2p proteins, compared with hC0-C2 proteins. The η values were calculated to be in the range of concentrations for the proteins used: 1.1 4PL (∗∗*p* < 0.001), 2.6 4PL (∗∗*p* < 0.001), and 0.4 4PL (∗∗*p* < 0.001) for hS2^R870H^, hS2^E924K^, and hS2^E930Δ^, respectively, for hC0-C2p (Fig. 4*F* and Table 3). The change in slope and η values for mutants to hC0-C2p further supports the SPBA data that mutant hS2 shows greater affinity for C0-C2 in the phosphorylated state, the opposite of that observed for hS2^Wt^. Altogether, both SPBA and ITC experiments confirmed that mutant hS2 proteins have increased affinity to hC0-C2 in the phosphorylated status by an increased slope and increased η between the two proteins.

### Mutant S2 proteins increased the k_tr_ in cardiac papillary fibers with phosphorylated cMyBP-C

**Figure 5:**
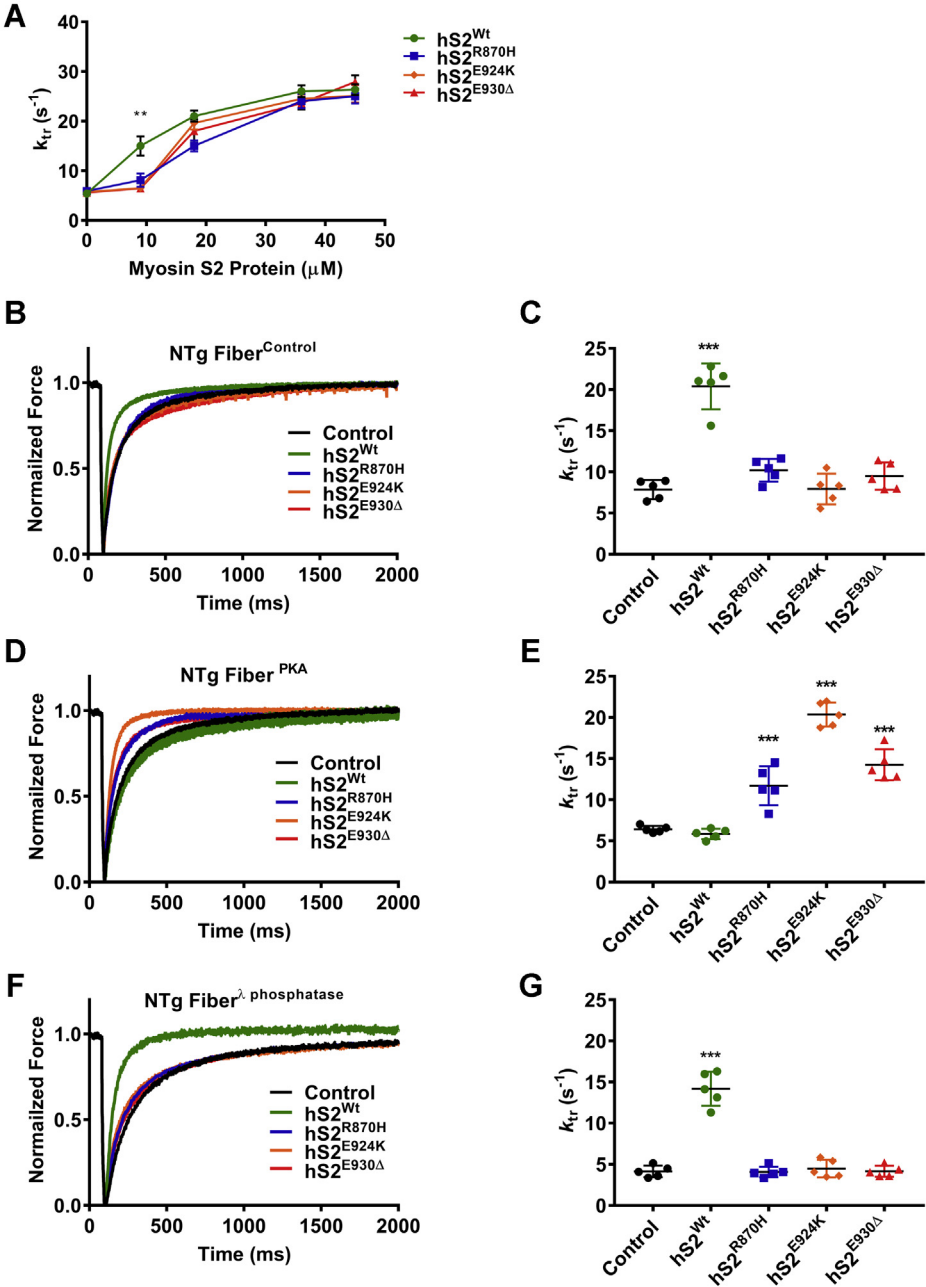
**Rate of force redevelopment (*k***_***tr***_**) was enhanced by mutant S2 proteins that were permeabilized in fibers**^**PKA**^**.***A*, raw *k*_*tr*_ traces for nontransgenic (NTg) fibers treated with all four S2 proteins with a concentration range from 9 to 45 μM, which determined that the 9 μM concentration of S2 proteins was sufficient to differentiate the effect of hS2^Wt^ to all mutant hS2 proteins. *B*, raw *k*_*tr*_ traces and *C*, values for fibers^control^ that were untreated (*black*) and treated with 9 μM hS2^Wt^ (*green*), hS2^R870H^ (*blue*), hS2^E924K^ (*orange*), and hS2^E930Δ^ (*red*) proteins. *D*, raw *k*_*tr*_ traces and *E*, values for fibers^PKA^ that were untreated (*black*) and treated with 9 μM hS2^Wt^ (*green*), hS2^R870H^ (*blue*), hS2^E924K^ (*orange*), and hS2^E930Δ^ (*red*) proteins. *F*, *k*_*tr*_ traces and *G*, values for fibers^λ-phosphatase^ that were untreated (*black*) and treated with 9 μM hS2^Wt^ (*green*), hS2^R870H^ (*blue*), hS2^E924K^ (*orange*), and hS2^E930Δ^ (*red*) proteins. Statistical analyses were performed by one-way ANOVA with Tukey’s multiple comparison test with single pooled variance. n = 5 fibers/3 mice (12 weeks old, FVB/N, mixed sex) were used at submaximal pCa 5.7 and sarcomere length 2.0 μM in the study. Data are expressed as the mean ± SEM. See Table 4 for analysis of main factors and interactions. ∗∗*p* < 0.01, and ∗∗∗*p* < 0.001 *versus* untreated control. hS2^E924K^, human recombinant proximal S2 protein with E924K mutation; hS2^E930Δ^, human recombinant proximal S2 protein with E930Δ mutation; hS2^R870H^, human recombinant proximal S2 protein with R870H mutation; hS2^Wt^, human recombinant proximal S2 WT protein; S2, subfragment 2 region.

We previously showed that preincubation of skinned cardiac papillary fibers treated with hS2^Wt^ could significantly increase the rate of force redevelopment (*k*_tr_) (33). In this assay, exogenous hS2 competes with endogenous cMyBP-C:myosin binding, increasing the number of myosin heads available for actin binding, hence accelerating *k*_tr_ (33). However, it remained unknown whether mutations in hS2 would also demonstrate this phenomenon. We hypothesized that reduced interaction of mutant hS2 with C0-C2 would prevent the increase in *k*_tr._ Accordingly, we measured maximal force generation at pCa 4.5 (Fig. S10, Table S2) and submaximal *k*_tr_ at pCa 5.7 using skinned cardiac papillary fibers (termed as fibers) from WT mice permeabilized with hS2 or its mutant proteins. In this experiment, we utilized WT permeabilized fibers treated in three different ways: (i) untreated control (fibers^control^), (ii) treated with PKA (fibers^PKA^), and (iii) treated with λ-phosphatase (fibers^λ-phosphatase^). These populations allowed us to probe the effect of hS2 peptides at different levels of sarcomeric protein phosphorylation, including endogenous cMyBP-C. Our previous study used hS2^WT^ at a concentration of 45 μM (33). However, this was 5-fold higher than the affinity observed by the SBPA and ITC assay. Therefore, the measurement of *k*_tr_ in fibers was performed with increasing dosage, ranging from 9 μM to 45 μM of hS2^Wt^ proteins. The increment of *k*_tr_ in fibers by hS2^Wt^ proteins was dose dependent (Fig. 5*A* and Fig. S11). From these results, it was evident for dosage above 9 μM, the differences were saturated over the *k*_*tr*_ for all the groups. Accordingly, we chose 9 μM of hS2 for all subsequent experiments to determine the exact differences between hS2^WT^ and HCM causing hS2 proteins.

Fibers^control^ had a baseline *k*_tr_ value of 7.86 ± 0.46 s^−1^; however, when treated with 9 μM hS2^Wt^ protein, a 2.6-fold increase in *k*_tr_ was observed (20.4 ± 1.24 s^−1^, ∗∗*p* < 0.001) (Fig. 5*B* and Table 4). When fibers^control^ were permeabilized with mutant S2 proteins, no effect on the *k*_tr_ was observed, with an average value of 9.22 ± 0.7 s^−1^ for all the mutants (Fig. 5*B* and Table 4), suggesting that mutant hS2 compared with hS2^Wt^ could not modulate cross-bridge formation and tension redevelopment. Next, fibers^PKA^ displayed a slight but nonsignificant reduction in the *k*_tr_ (6.42 ± 0.16 s^−1^), consistent with previous work (48, 49, 50). Interestingly, incubating fibers^PKA^ with hS2^Wt^ failed to accelerate the *k*_tr_ as observed in fiber^control^ experiments (5.84 ± 0.3 s^−1^) (Fig. 5*C* and Table 4). However, treatment of fibers^PKA^ with mutant hS2 resulted in a significant increase in the *k*_tr_ for hS2^R870H^ (11.69 ± 1.06 s^−1^, ∗∗*p* < 0.001), hS2^E924K^ (20.36 ± 0.66 s^−1^, ∗∗*p* < 0.001), and hS2^E930Δ^ (14.25 ± 0.85 s^−1^, ∗∗*p* < 0.001), compared with untreated control fibers^PKA^. Fibers^λ-phosphatase^ displayed a significantly slower *k*_tr_ than fibers^control^ (4.13 ± 0.32 s^−1^, ∗∗*p* < 0.001). Again, we observed divergent effects of S2^WT^ (*k*_tr_ =14.17 ± 0.92 s^−1^, ∗∗*p* < 0.001) and mutant hS2, which did not affect the *k*_tr_ in phosphatase-treated fibers: hS2^R870H^ (4.07 ± 0.29 s^−1^), hS2^E924K^ (4.48 ± 0.48 s^−1^), or hS2^E930Δ^ (4.16 ± 0.30 s^−1^). The *k*_tr_ values for all the experiments are summarized in Table 4. Taken together, our data further demonstrate the differential binding of WT and mutant hS2 under basal and phosphorylated conditions. Importantly, these data suggest different physiologic responses to adrenergic stimulation when hS2 mutations are present.

**Table 4:** Rate of force redevelopment (*k*_tr_, s^−1^) for NTg fibers permeabilized with hS2 proteins

hS2 Proteins	n	Fibers^control^	Fibers^PKA^	Fibers^λ-phosphatase^
Control	5	7.86 ± 0.46	6.42 ± 0.16	4.13 ± 0.32^¶¶¶,††^
hS2^Wt^	5	20.4 ± 1.24∗∗∗	5.84 ± 0.29^¶¶¶^	14.17 ± 0.92∗∗∗^,¶¶¶,†††^
hS2^R870H^	5	10.22 ± 0.62^§§§^	11.69 ± 1.06∗∗∗^,§§§^	4.07 ± 0.29^§§§,¶¶¶,†††^
hS2^E924K^	5	7.94 ± 0.83^§§§^	20.36 ± 0.6∗∗∗^,§§§,¶¶¶^	4.49 ± 0.48^§§§,†††^
hS2^E930Δ^	5	9.51 ± 0.75^§§§^	14.25 ± 0.85∗∗∗^,§§§,¶¶^	4.16 ± 0.30^§§§,¶¶¶,†††^

Abbreviation: NTg, nontransgenic.

Statistical significance was calculated by ordinary one-way ANOVA with Tukey’s multiple comparison test with single pooled variance. ± values are indicative of the SEM for observed *k*_*tr*_ values. ‘n’ is equal to the number of skinned papillary fibers used for the measurements, where one fiber per animal was utilized (12-week-old NTg mice, FVB/N and mixed sex) and measured *k*_*tr*_ values at submaximal pCa 5.7 and sarcomere length 2.0 μM. See Table 7 for analysis of main factors and interactions.

∗*p* < 0.05, ∗∗*p* < 0.01, ∗∗∗*p* < 0.001 *versus* control.

^§^*p* < 0.05, ^§§^*p* < 0.01, ^§§§^*p* < 0.001 *versus* hS2^Wt^.

^¶^*p* < 0.05, ^¶¶^*p* < 0.01, ^¶¶¶^*p* < 0.001 *versus* respective Fibers^control^.

^†^*p* < 0.05, ^††^*p* < 0.01, ^†††^*p* < 0.001 *versus* respective Fibers^PKA^.

**Figure 6:**
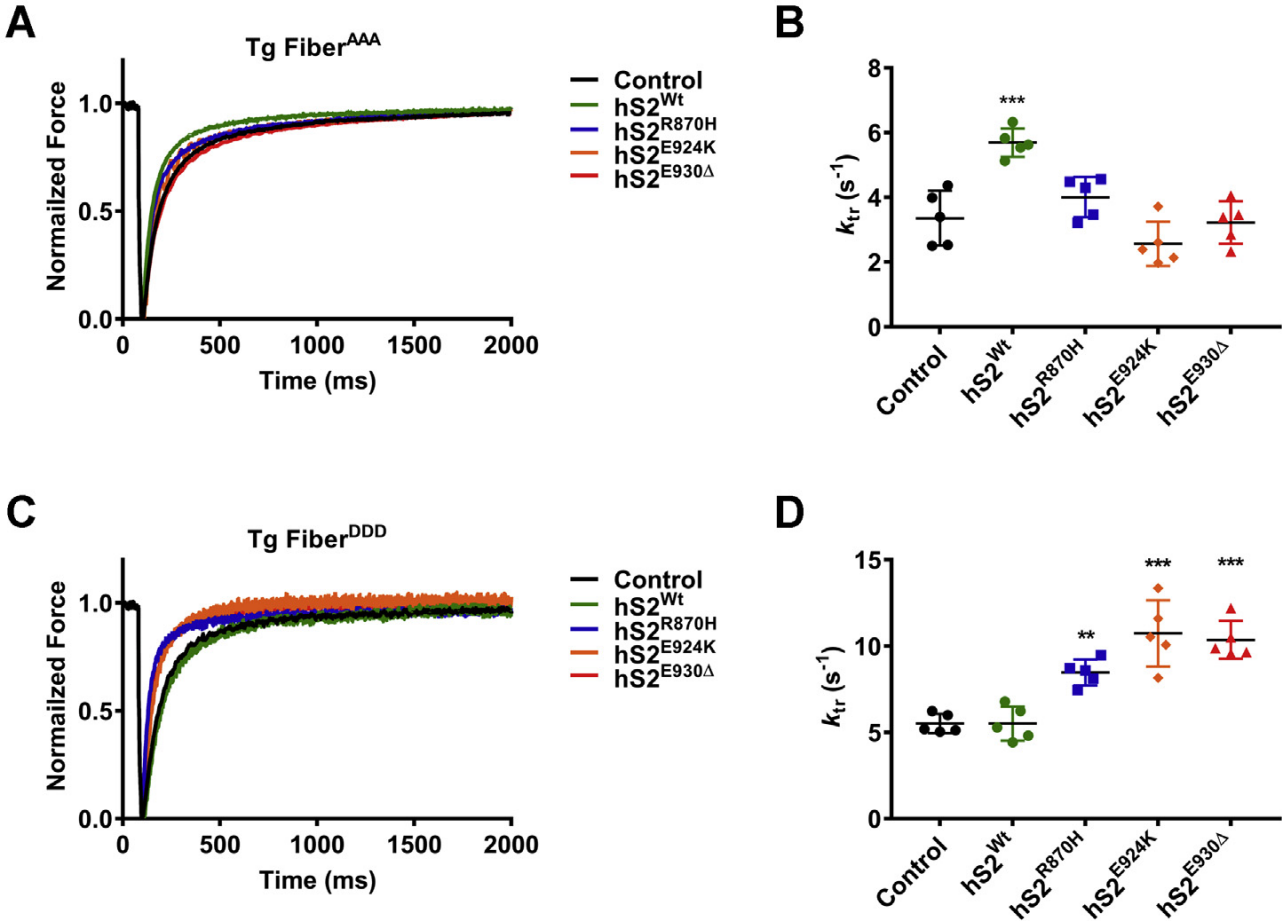
**Rate of force redevelopment (*k***_**tr**_**) was elevated by mutant hS2 proteins in fibers expressing cMyBP-C DDD in transgenic (Tg) protein.***A*, raw *k*_tr_ traces and *B*, values for fibers that expressed cMyBP-C AAA without treatment (*black*) and fibers treated with 9 μM hS2^Wt^ (*green*), hS2^R870H^ (*blue*), hS2^E924K^ (*orange*), and hS2^E930Δ^ (*red*) proteins. *C*, raw *k*_*tr*_ traces and *D*, values for fibers that expressed cMyBP-C DDD without treatment (*black*) and treated with 9 μM hS2^Wt^ (*green*), hS2^R870H^ (*blue*), hS2^E924K^ (*orange*), and hS2^E930Δ^ (*red*) proteins. Statistical analyses were performed by one-way ANOVA, and Tukey’s pooled multiple variance test was used to calculate the significance between the *k*_tr_ values. n = 5 fibers/3 mice (12 weeks old, FVB/N, mixed sex), where n is each fiber treated with a single concentration of myosin S2 protein and measured *k*_*tr*_ at submaximal pCa 5.7 and sarcomere length 2.0 μM. Data are expressed as the mean ± SEM. See Table 5 for analysis of main factors and interactions. ∗∗*p* < 0.01, and ∗∗∗*p* < 0.001 *versus* untreated controls. AAA, phospho-ablated cMyBP-C or phospho-ablation; cMyBP-C, cardiac myosin-binding protein-C; DDD, phospho-mimetic cMyBP-C; hS2^E924K^, human recombinant proximal S2 protein with E924K mutation; hS2^E930Δ^, human recombinant proximal S2 protein with E930Δ mutation; hS2^R870H^, human recombinant proximal S2 protein with R870H mutation; hS2^Wt^, human recombinant proximal S2 WT protein; S2, subfragment 2 region.

The mutant hS2 proteins were further analyzed by measuring *k*_*tr*_ in fibers from mice expressing phospho-ablated cMyBP-C (AAA) (32) and phospho-mimetic cMyBP-C (DDD) (25). As expected, hS2^Wt^ protein increased the *k*_tr_ in AAA papillary fibers (control *k*_*tr*_ = 3.36 ± 0.37 s^−1^, hS2^Wt^ *k*_*tr*_ = 5.69 ± 0.20 s^−1^, ∗∗∗*p* < 0.001), but not in the DDD fibers (control *k*_*tr*_ = 5.52 ± 0.25 s^−1^, hS2^Wt^ *k*_*tr*_ = 5.3 ± 0.44 s^−1^). Conversely, mutant hS2 proteins were able to increase the *k*_*tr*_ in DDD fibers (control *k*_*tr*_ = 5.52 ± 0.25 s^−1^, hS2^R870H^ *k*_*tr*_ = 8.48 ± 0.34 s^−1^, ∗∗∗*p* < 0.001; hS2^E924K^ *k*_*tr*_ = 10.74 ± 0.85 s^−1^, ∗∗∗*p* < 0.001; hS2^E930Δ^ *k*_*tr*_ = 10.36 ± 0.49 s^−1^, ∗∗∗*p* < 0.001), but not in AAA fibers (control *k*_*tr*_ = 3.36 ± 0.37 s^−1^, hS2^R870H^ *k*_*tr*_ = 4 ± 0.27 s^−1^, hS2^E924K^ *k*_*tr*_ = 2.57 ± 0.31 s^−1^, hS2^E930Δ^ *k*_*tr*_ = 3.22 ± 0.29 s^−1^) (Fig. 6 and Table 5). Overall, in DDD fibers, the *k*_tr_ was unaffected by hS2^Wt^ protein; however, mutant hS2 proteins accelerated *k*_tr_ with mutant hS2 proteins. The results from the *in situ* fiber experiments corroborated our results from SPBA and ITC experiments, which showed that increased affinity of mutant hS2 to phosphorylated cMyBP-C would allow for the faster *k*_*tr*_ observed in mutant hS2-treated fibers^PKA^ and fibers^DDD^. The data overall suggest that binding between N-terminal cMyBP-C and proximal myosin S2 is based on phosphorylation of cMyBP-C and that this interaction could alter cross-bridge kinetics in the heart.

**Table 5:** The *k*_tr_ for fibers of AAA and DDD transgenic mice treated with hS2 proteins

hS2 Proteins	n	AAA-Tg fiber	DDD-Tg fiber
Control	5	3.36 ± 0.37	5.52 ± 0.25^¶^
hS2^Wt^	5	5.69 ± 0.20∗∗∗	5.3 ± 0.44
hS2^R870H^	5	4 ± 0.27^§§^	8.48 ± 0.34∗∗^,§§,¶¶¶^
hS2^E924K^	5	2.57 ± 0.31^§§§^	10.74 ± 0.85∗∗∗^,§§§,¶¶¶^
hS2^E930Δ^	5	3.22 ± 0.29^§§§^	10.36 ± 0.49∗∗∗^,§§§,¶¶¶^

Abbreviation: Tg, transgenic.

Statistical significance was calculated by ordinary one-way ANOVA with Tukey’s multiple comparison test with single pooled variance. ‘n’ is equal to the number of skinned papillary fibers used for each measurement, where one papillary fiber was utilized per animal (12 weeks old, FVB/N, and mixed sex). ± values are the SEM for measured *k*_*tr*_ values at submaximal pCa 5.7 and sarcomere length 2.0 μM. See Table 7 for analysis of main factors and interactions.

∗*p* < 0.05, ∗∗*p* < 0.01, ∗∗∗*p* < 0.001 *versus* control.

^§^*p* < 0.05, ^§§^*p* < 0.01, ^§§§^*p* < 0.001 *versus* hS2^Wt^.

^¶^*p* < 0.05, ^¶¶^*p* < 0.01, ^¶¶¶^*p* < 0.001 *versus* respective AAA-Tg fibers.

### Mutant S2 proteins are susceptible to structural disorder by a chaotropic agent

**Figure 7:**
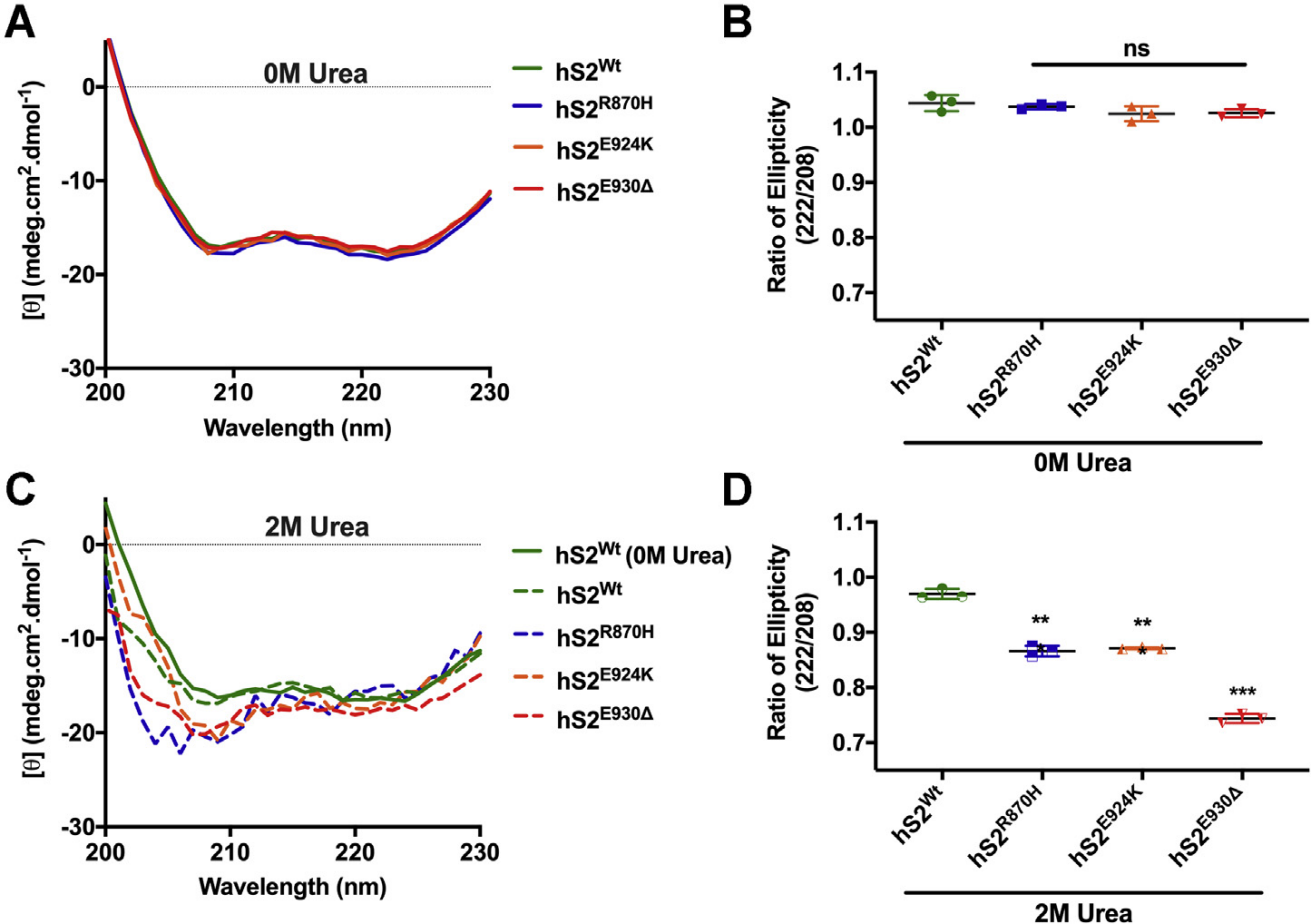
**Mutant S2 proteins were unable to keep coiled coil structure intact under high-salt buffer conditions.***A*, the α-helical coiled coil spectra for hS2^Wt^ (*green*), hS2^R870H^ (*blue*), hS2^E924K^ (*orange*), and hS2^E930Δ^ (*red*) proteins in the absence of urea. *B*, the ratio of ellipticity (222/208) for S2 in a low-salt buffer with no change in coiled coil structure. *C*, the effect of 25% or 2 M urea on α-helical coiled coil spectra for hS2^Wt^ (*green*), hS2^R870H^ (*blue*), hS2^E924K^ (*orange*), and hS2^E930Δ^ (*red*) proteins. *D*, the ratio of ellipticity (222/208) for hS2 in 2 M urea buffer with a significant change in coiled coil structure to independent α-helices. Statistical analyses were performed by one-way ANOVA with Tukey’s multiple comparison test with single pooled variance (n = 3). Data are expressed as the mean ± SEM. See Table 6 for analysis of main factors and interactions. ∗∗*p* < 0.01, and ∗∗∗*p* < 0.001 *versus* hS2^Wt^ controls. hS2, human cardiac myosin S2; hS2^E924K^, human recombinant proximal S2 protein with E924K mutation; hS2^E930Δ^, human recombinant proximal S2 protein with E930Δ mutation; hS2^R870H^, human recombinant proximal S2 protein with R870H mutation; hS2^Wt^, human recombinant proximal S2 WT protein; ns, non-significant; S2, subfragment 2 region.

Myosin S2 is an extensive coiled coil comprising two α helices wound around each other by hydrophobic interactions between the heptad repeats of each α-helix (51). A point mutation in hS2 could affect α-helical structure by disrupting the heptad repeat, thus impacting the stability of the hS2 coiled coil (52). Hence, we measured the stability of the hS2^Wt^ α-helix *versus* mutant hS2 by CD. The ratio of ellipticity at 222/208 nm ≥ 1.0 is representative of a coiled coil structure, whereas a ratio ≤0.9 represents a much more independent α-helical structure (53). In low salt buffer, no significant changes were observed between hS2^Wt^ and mutant S2 coiled coil structure, with all hS2 forming a coiled coil (Fig. 7, *A* and *B*, and Table 6). Next, the CD spectra for hS2^Wt^ and mutant hS2 were measured in presence of 2 M urea in low-salt buffer (54). Upon urea treatment, the ratio of ellipticity at 222/208 nm for hS2^Wt^ was at 0.97, still maintaining a coiled coil structure. However, the ratio for 222/208 nm was significantly decreased across the mutant hS2 proteins at 0.87 (∗∗*p* < 0.001), 0.86 (∗∗*p* < 0.001), and 0.74 (∗∗*p* < 0.001) for hS2^R870H^, hS2^E924K^, and hS2^E930Δ^ proteins, respectively (Fig. 7, *C* and *D* and Table 6). The decreased ratio for mutant S2 proteins in the presence of urea demonstrated the loss of their coiled coil and the formation of a more isolated α-helical structure, whereas no change was observed for the hS2^Wt^ coiled coil structure in the presence of a denaturant. Thus, hS2 containing these mutations are more readily susceptible to structural changes, leading to instability of the myosin hS2 coiled coil. A stable myosin S2 produced less-effective force; hence, an unstable myosin S2 coiled coil could increase the amount of force produced in muscle fibers, resulting in the hypercontractile phenotype observed in patients who carry the mutations examined in this study (55).

**Table 6:** Ellipticity (mdeg.cm^2^.dmol^−1^) values observed for hS2 proteins in low-salt buffer and 2 M urea buffer at 208 and 222 nm by CD spectra

hS2 Proteins	n	0 M urea			2 M urea		
		208 nm	222 nm	222/208	208 nm	222 nm	222/208
hS2^Wt^	3	−16.95 ± 0.27	−17.63 ± 0.14	1.04 ± 0.01	−16.95 ± 0.19	−16.43 ± 0.13	0.97 ± 0.01^¶¶¶^
hS2^R870H^	3	−17.52 ± 0.06	−18.18 ± 0.11	1.04 ± 0.002^§§§^	−20.06 ± 0.04	−17.37 ± 0.13	0.86 ± 0.01^§§§,¶¶¶^
hS2^E924K^	3	−16.87 ± 0.60	−17.20 ± 0.48	1.02 ± 0.01^§§§^	−19.56 ± 0.21	−17.06 ± 0.16	0.87 ± 0.001^§§§,¶¶¶^
hS2^E930Δ^	3	−16.83 ± 0.32	−17.26 ± 0.26	1.03 ± 0.004^§§§^	−20.46 ± 0.03	−15.03 ± 0.12	0.74 ± 0.005^§§§,¶¶¶^

Statistically significant values were calculated by one-way ANOVA calculated with Tukey’s multiple comparison test with single pooled variance. ‘n’ is equal to the number of independent experiments performed for each CD measurement. ± values are for the SEM for reported values. See Table 7 for analysis of main factors and interactions.

^§^*p* < 0.05, ^§§^*p* < 0.01, ^§§§^*p* < 0.001*versus* hS2^Wt^ + 2M Urea.

^¶^*p* < 0.05, ^¶¶^*p* < 0.01, ^¶¶¶^*p* < 0.001 *versus* respective 0M Urea.

## Discussion

### Loss of interaction between myosin S2 and dephosphorylated N-terminal cMyBP-C

The present study determined how mutant myosin S2 protein interacts with the C0-C2 region of cMyBP-C in the presence and absence of C0-C2 phosphorylation. Three HCM-causing mutations (R870H, E924K, and E930Δ) that reside within the proximal S2 of both α-myosin heavy chain and β-MyHC (amino acids 838–963) were chosen for the study. The binding affinity and binding capacity between hS2 and the N-terminal C0-C2 region of cMyBP-C was determined by the SPBA and confirmed using the ITC assay.

Both these methods revealed that all three hS2 mutants had reduced binding capacity for dephosphorylated C0-C2, with no change in their binding affinity, when compared with hS2^Wt^. The binding variables in our ITC experimental findings were similar to those of ITC experiments performed earlier using only the C1-C2 domains of cMyBP-C (5, 24) and supported SBPA results that mutant S2 binding to N-terminal cMyBP-C domains is highly diminished under basal conditions. We then tested the effect of this reduced binding of mutant S2 to C0-C2 on cross-bridge kinetics. When S2^Wt^ was added endogenously to skinned fibers, it accelerated the *k*_*tr*_ (33). The faster *k*_*tr*_ is thought to result from the binding of recombinant S2^Wt^ protein to endogenous cMyBP-C in the fibers, thereby releasing its inhibitory interaction with endogenous myosin S2, enabling S1 to engage actin thin filaments more rapidly. Using this indirect assessment of the effect of myosin S2 and C0-C2 interaction on the *k*_*tr*_, we showed mutant S2 did not increase the *k*_*tr*_ in papillary fibers. This further demonstrates that mutant S2 proteins interact minimally with cMyBP-C and that they subsequently cannot compete with endogenous myosin interaction with cMyBP-C *in situ*. These findings suggest that endogenous myosin containing hS2 mutations would have only limited interaction with the N-terminal region of cMyBP-C, which can explain the hypercontractile phenotype associated with these mutations in the S2 region.

Loss of this interaction would inhibit myosin heads from adopting the SRX state of myosin, thereby increasing the pool of myosin available for cardiac contraction, resulting in sarcomeric hypercontractility (43). S2 plays a critical structural role in sequestering myosin heads in the SRX, and we cannot exclude a direct effect of the mutations on binding between myosin S1-S2 in addition to cMyBP-C binding (33, 37, 56). Force spectroscopy and *in vitro* motility assays have additionally shown that dephosphorylated N-terminal cMyBP-C acts as a load to stabilize the S2 coiled coil, thus decreasing the amount of S1 heads available to move actin filaments (15, 55) In sum, these results suggest that S2, along with dephosphorylated N-terminal cMyBP-C, renders the S1 heads inactive with respect to reduced cross-bridge formation. Thus, in the case of myosin containing S2 mutations, the ability of cMyBP-C to restrict the myosin heads would be diminished, leading to pathological perturbation of both the contraction and relaxation of the heart.

### Mutant S2 preferentially interacts with phosphorylated N-terminal cMyBP-C

cMyBP-C is highly phosphorylated under normal conditions and undergoes dephosphorylation as a result of cardiac stress, including aging, and heart failure (12, 44, 57). Furthermore, HCM caused by mutations in *MYH7* exhibit reduced levels of cMyBP-C phosphorylation (58, 59). This reduced cMyBP-C phosphorylation may result from the depressed β-AR response observed in patients with HCM (57, 60, 61, 62). Hence, we tested the interaction between mutant S2 and phosphorylated C0-C2 proteins. The binding between S2^Wt^ and C0-C2p was attenuated, as measured by the SPBA and ITC assay, which could potentially cause decreased myofilament Ca^2+^ sensitivity and accelerated contractility. The SPBA revealed a lower *B*_max_ and higher *K*_*d*_ between S2^Wt^ and C0-C2p proteins, as previously reported (5). Furthermore, the binding studies revealed that significantly increased amount of mutant hS2 proteins were required to saturate with C0-C2p proteins yet had an increased affinity toward C0-C2p protein. This effect was validated independently by ITC with similar results. Strikingly, tighter binding affinity between mutant S2 and C0-C2p was revealed by both the SPBA and ITC assay. Next, we assessed the functional effect of the increased affinity of mutant S2 on the *k*_*tr*_ in fibers^PKA^. Interestingly, although S2^Wt^ proteins had no impact on the submaximal *k*_*tr*_ in fibers^PKA^, mutant S2 proteins accelerated the *k*_*tr*_. This change was validated by measuring the *k*_*tr*_ in phospho-mimetic DDD fibers treated with mutant S2, compared with control fibers. We also performed additional experiments where we increased the dephosphorylation in cMyBP-C with the help of λ-phosphatase and found that S2^Wt^ but not mutant S2 was able to increase the *k*_*tr*_. The fiber experiment showed that mutant S2 could only affect the *k*_*tr*_ in an increased or heightened state of cMyBP-C phosphorylation in fibers. When fibers are treated with PKA, cMyBP-C is maximally phosphorylated at Ser-273, Ser-282, and Ser-302. As a result, N-terminal region of cMyBP-C loses its interaction with myosin and may prefer interaction with thin filaments (24). At the same time, however, cMyBP-C will compete with myosin for actin binding. This may explain why PKA treatment does not increase the *k*_*tr*_ for fibers^PKA^, allowing actin interaction with myosin and cMyBP-C to be in a competitive state. The addition of mutant S2 proteins would occupy the population of maximally phosphorylated cMyBP-C. This, in turn, creates a state in which cMyBP-C can interact with neither actin thin filaments nor myosin thick filament, thus allowing myosin to freely occupy actin sites, resulting in an increased *k*_tr_. In line with this theory, studies on the absence of N-terminal cMyBP-C (63) or KO of cMyBP-C (64) have reported that the *k*_*tr*_ is much elevated compared with the control. Together, these data could indicate that during adrenergic stimulation, in which cMyBP-C phosphorylation is at its highest, these mutations in the S2 region of β-MyHC would increase the interaction with the N-terminal domains of cMyBP-C (65). This interaction at a time when β-MyHC would normally not interact with the N-terminus of cMyBP-C may inhibit myosin heads from actin interaction, thereby could lead to a reduced adrenergic response. Over time, this has been demonstrated to lead to cardiac stress and onset of disease (60, 61, 62).

### Mutant S2 is structurally unstable

CD spectra of S2^Wt^ and mutant S2 in low-salt buffer showed that the coiled coil structure was intact in all S2 proteins. The ratio of ellipticity at 222/208 was higher than 1 for all S2 proteins. However, CD spectra of S2^Wt^ and mutant S2 in the presence of the denaturant, urea, at low concentrations showed that S2^Wt^ had its coiled coil structure intact, whereas the mutant hS2 proteins showed greater disorganization. Previously, by laser temperature-jump studies, an altered S2 coiled coil with increased dissociation results in elevated contractile kinetics in the sarcomere (66). The S2 region in myosin is an extensive coiled coil structure, which itself is unstable and could be disorganized to affect actomyosin interaction (67). An antibody to the whole myosin S2 coiled coil dampened the contractile force in muscle fibers, and an opposite effect was observed when the S2 coiled coil structure was melted during laser temperature-jump studies, leading to increased contractile force (68). Hence, a mutation that increases the susceptibility of the S2 coiled coil instability could promote hypercontractility by increasing the availability of myosin heads to actin thin filaments. The propagation of contractile force in the C zone of the sarcomere is dysregulated because of increased affinity of phosphorylated cMyBP-C to S2 (69). This compensation for producing force could be aided by an unstable S2 coiled coil. The relaxation would be impaired as well because dephosphorylated cMyBP-C would not be able to bind S2 to return active myosin heads to the inactive SRX state. Because phosphorylation of cMyBP-C is reduced by aging (44), S2 would have increased binding affinity with cMyBP-C, leading to severe HCM and heart failure.

## Perturbed coiled-coil in mutant S2 as a possible cause of hypercontractility and HCM

The S2 region is the hotspot for HCM mutations (9). Based on the observations from the present study from the perspective of pathogenic HCM, the mutant S2 proteins have a loosened coil-coil structure which would explain the easy access and stretch of the myosin heads, as well as the availability of more myosin heads for the formation of increased actomyosin interactions and cross-bridges, thereby enhancing contractility (70). This is because the R870H, E924K, and E930Δ mutations localize in the core positions a, e, and d of the coiled coil, respectively (Fig. S12). That conformation might perturb interhelical bridge formation and possibly affect coiled-coil stability and structure (71), thereby limiting the ability of myosin heads to adopt the SRX state, where these heads fold back to interact with their myosin S2 region (72). The upshot of this inability to form the SRX would be a greater fraction of myosin available to bind actin and, hence, an increase in the ensemble force. Recent X-ray diffraction studies have shown that peak force during contraction and generation of contractile force is primarily associated with the cMyBP-C zone of the sarcomere (50). Consequently, S2 mutations may result in activation of contractile force by myosin in zones other than C-zone of sarcomere. Because the other zones are void of cMyBP-C, S2 is unloaded, and it may be that the mutation in S2 could increase the availability of S1 heads by reducing coiled coil stability. Next, the question is how these mutations differentially regulate the actomyosin interactions in the presence of cMyBP-C phosphorylation. Normally, cMyBP-C is highly phosphorylated in the M domain under baseline conditions and regulates actomyosin interactions by interacting with myosin S2 regions (73). Dephosphorylated cMyBP-C strongly interacts with myosin S2, which was shown using C0-C2 recombinant proteins *in vitro*. In addition, the level of cMyBP-C is increasingly dephosphorylated during heart failure, which contributes to decreased force generation of cardiac muscles. In contrast, phosphorylated cMyBP-C shows significantly reduced interaction with myosin S2, allowing actomyosin interactions to enhance contractility under β-AR stimulation (74). By the same mechanism, mutants in S2 regions exhibit reduced binding with cMyBP-C, promoting hypercontractility under baseline conditions. However, upon β-AR stimulation, mutant S2 shows enhanced interactions with phosphorylated cMyBP-C in the present study using C0-C2 regions. We expect that this enhanced interaction reduces maximal force generation as a natural compensatory mechanism to regulate force. This may explain why moderate exercise significantly improves the clinical outcome of HCM phenotype (75). Aging, however, and the presence of secondary cardiovascular stress applied by altered adrenergic response drive HCM pathogenesis (61, 62, 76). Therefore, modulating S2 and cMyBP-C interactions *via* M-domain phosphorylation would be a potential treatment strategy to improve the HCM phenotype.

## Limitations, summary, and future directions

The major limitation of our studies involves the nature of mutant S2 interactions with S1 in the off state, which was not studied here. For example, it was shown that hS2^R870H^ loses its interaction with the mesa region, the proximal upper region of S1 (26, 42). In the presence of S1, a competitive binding analysis with mutant S2 and C0-C2 phosphorylation would be a potential set of future experiments. However, taken together, our experiments do confirm that mutations in S2 result in the loss of S2 interaction with dephosphorylated cMyBP-C. Another limitation involves the *k*_*tr*_, which was unaffected in fibers treated with PKA. Some studies show a modest increase in the *k*_*tr*_ (30, 77, 78), but a review of the literature also suggests that PKA had no effect on the *k*_*tr*_, although accompanied by a reduction in maximal force produced (48, 49, 50, 79, 80, 81). However, S2 mutations showed increased affinity to phosphorylated cMyBP-C, unlike S2 WT. Furthermore, we showed that mutations in S2 make the coiled coil highly susceptible to instability when challenged with an entropy-inducing change. Other major outcomes of this study show that interaction between S2 and cMyBP-C is a critical regulator of contractility, both basally and during adrenergic stimulation. When the interaction is affected by mutations, it would detrimentally affect the rate of cross-bridge formation. We have demonstrated that the interaction between S2 and C0-C2 can be challenged with external proteins to affect the cross-bridge formation. This study opens a novel way to target cardiac force generation by competing S2/C0C2 interaction. The extended study of this interaction will help formalize new therapeutic agents to improve cardiac diseases, such as HCM and heart failure, as the interaction is confined to the C zone of the sarcomere where maximal force has been generated (82). If therapeutic agents could improve the binding of S2 to cMyBP-C in case of HCM mutations, it would promote myosin head sequestration, rectifying hypercontractility. On the other hand, if therapeutic agents could block the interaction of S2 to cMyBP-C, more myosin heads would be available for contraction and thus increase contractility in diseases such as dilated cardiomyopathy and heart failure. Our studies lay the groundwork for the study of these two interacting proteins in more depth. Accordingly, future studies will expand the current findings by using animal models and performing preclinical studies to treat HCM.

## Experimental procedures

### Recombinant proteins

*Escherichia coli* (BL21) cultures with the *pET*^*28a+*^ vector (Novagen) containing the cDNA insert for all the human *MYH7*; myosin S2 (proximal 126 amino acids, from 838 to 963 codons, UniProt ID: P12883) proteins and human *MYBPC3*; and C0-C2 protein (452 amino acids, from 1 to 452 codons, UniProt ID: Q14896) were used in the study to perform the interaction studies (Fig. 1*B*). Human myosin S2 mutations of R870H, E924K, and E930Δ were introduced into the WT myosin S2 cDNA using the Q5 site-directed mutagenesis kit (New England Biolabs) and specific primers to introduce the mutations in the WT human myosin S2 cDNA (Table S3). The sequences of each construct were confirmed by Sanger sequencing. Each myosin S2 construct contained a C-terminal 6x His-tag, whereas C0-C2 proteins contained an N-terminal 6x His-tag. The recombinant proteins were purified from bacterial cultures by cometal affinity columns (Catalog 63550, TALON Cobalt Resin, Clontech). Densitometry analysis was performed on all bands observed for purified product on the Coomassie SDS-PAGE gel to give the overall purity of the recombinant protein (Fig. S3). Purified proteins were verified by performing Western blots using primary antibodies to detect His-tag, myosin S2 and C0-C2 protein. In addition, we utilized recombinant mouse cMyBP-C, C0-C2 protein containing three alanine (C0-C2^AAA^, phospho-ablation) or three aspartate (C0-C2^DDD^, phospho-mimetic) residues in place of serine-273, serine-282, and serine-302. These constructs have been previously described (18, 33) and were utilized to validate the SPBA binding experiments in the present studies.

### Phosphorylation of hC0-C2 by PKA

hC0-C2 proteins were phosphorylated using the PKA catalytic subunit from bovine heart (Sigma, Cat. # P2645). PKA (0.04 units) was used per 1 μg of hC0-C2 protein in PBS containing the additions of 1 mM ATP, 6 mM DTT, and 10 mM magnesium chloride at pH 7.4. Phosphorylation reactions were carried out for 90 min at room temperature (RT) with agitation at ten rotations per minute on a horizontal shaker. hC0-C2 phosphorylation was verified by Western blots (Fig. S4A) using custom-made phospho-specific antibodies (1:2500 p273, 1:10,000 p282, and 1:2500 p302) to hC0-C2 protein (21).

### SPBA *in vitro*

The SPBA was performed to determine the level of interactions between hS2 and hC0-C2. To perform this assay and validate the data, two different primary antibodies were used in the study. The first primary antibody was against human myosin S2 region that was raised in rabbits. Using the recombinant hS2-specific peptide (838-PLLKSAEREKEMASMKEE-855 codons), polyclonal antibodies were raised (ProSci) and validated in the present study (Fig. S6). The detection limits for myosin S2 were determined by adsorbing increasing concentration of myosin S2 from 0 to 6000 μM and verified by Western blot analysis. The micromolar range for detection of myosin S2 was determined to be at a maximum of 60 μM with the median at 17 μM (Fig. S7). The second primary antibody was against the N region of hC0-C2 (anti-rabbit 2–14 antibody, ProSci), the first 14 residues (21). All the SPBA experiments were performed with 20 μM concentration of S2 protein to yield measurable fluorescence intensity without saturation. For the experiment, 20 μM of C0-C2 protein were adsorbed onto microtiter plates overnight at 4 °C and the next day subjected to increasing (0–20 μM) concentrations of recombinant myosin S2 protein with or without mutations. The amount of myosin S2 bound was then detected by the novel polyclonal anti-S2 antibody (1:250), followed by a secondary Alexa Fluor 568 anti-rabbit antibody (1:5000, Cat. # A11036, Fisher Scientific) and detected by a Cytation 5 plate reader with excitation at 568 nm and emission at 619 nm. All binding experiments were repeated three times with a minimum of three replicates per experiment. The fluorescence intensity observed for each myosin S2 concentration was normalized to the intensity observed at the maximal myosin S2 concentration. The data were then fit with one-phase association curve in GraphPad Prism to yield the maximal affinity concentration (*B*_max_) and the rate of equilibrium *K*_*d*_ values. The *B*_max_ and *K*_*d*_ across all the experiments were compared and reported as relative *B*_max_ and *K*_*d*_ in micromolar concentration.

### ITC assay

ITC experiments were carried out to validate the SPBA experiments to determine the interactions between hS2 and hC0-C2, following previously published protocols using the MicroCal VP-ITC 2000 calorimeter (5, 24). Briefly, 20 μM of hC0-C2 protein was titrated against 350 μM of S2 protein in PBS at 20 °C. Protein solutions were degassed before titration. Diluent and titrant controls were performed to outline the true thermal interaction between recombinant hS2 and hC0-C2 proteins. Ten microliter of titrant injection at an interval of 180 s for a total of 25 injections was carried out in each experiment. Then, a titration speed of 351 rpm was carried out to ensure binding. ΔH, *K*_*d*_, and η were calculated by fitting the amount of heat produced at each titration and the η of hS2 and hC0-C2 to a four-parameter logistic curve (4PL) Y = y0 + (xˆHillslope)∗(Top-Bottom)/(xˆHillSlope+EC50ˆHillSlope) model in GraphPad Prism 7.

### CD experiments

To determine whether mutations affect S2 coiled-coil structure, CD experiments were performed as described previously (83). Recombinant hS2 protein was dialyzed overnight at 4 °C in 100 mM sodium carbonate, pH 8.0. The dialyzed proteins were diluted to 0.5 mg/ml with fresh 100 mM sodium carbonate, pH 8.0. Then, S2 protein was added to a quartz cuvette at 25 °C to measure the alpha helical content by the change in ellipticity of plane polarized light at 208 and 222 nm in a PerkinElmer spectrophotometer (Model 215, Akron, Ohio). To compare the stability of mutant hS2 coiled coils to WT hS2 control, 2 M urea were added to dialyzed recombinant proteins, and CD spectra were measured again. The ratio of ellipticity at 222:208 nm was compared between urea denatured and nontreated recombinant S2 proteins to compare the change in the tertiary structures of mutant S2 to WT S2.

### Maximal force and the k_tr_

Cardiac papillary fibers were dissected from the left ventricles of 12- to 14-week-old FVB/N mice (mixed sex), either WT or transgenic mice expressing AAA (32) and DDD (25). All experiments were conducted under institutional guidelines and approved by the University of Cincinnati Animal Care and Use Committee. Dissection and permeabilization of skinned papillary fibers with hS2 protein protocols were followed as described previously (33). Briefly, papillary fibers were skinned overnight at 4 °C in 1% Triton X-100 in the relaxing buffer (55.74 mM potassium propionate, 7 mM ethylene glycol bis (2-aminoethyl) tetraacetic acid, 100 mM N,N-bis(2-hydroxyethyl)-2-amino ethanesulfonic acid, 0.02 mM calcium chloride, 5.5 mM magnesium chloride, 5 mM DTT, 15 mM creatine phosphate, and 4.7 mM adenosine triphosphate with pH adjusted to 7.0 with 4 M potassium hydroxide and ionic strength maintained at 180 with potassium propionate) with pCa 9.0 as specified in the study (33). Maximal calcium solution (pCa 4.5) had a chemical makeup similar to that of the relaxing buffer, except it had 7 mM calcium chloride. Submaximal calcium solution (pCa 5.7) was prepared by mixing 83.5% of pCa 4.5 and 16.5% of pCa 9.0 solutions. The skinned papillary fibers approximately 150 microns thick were clipped between aluminum T-clips and then suspended over the length controller and force transducer of the Aurora Scientific 1400A system. The sarcomere length in skinned papillary fibers was maintained at 1.9 to 2.0 μM using the 600A digital controller and high-speed video sarcomere length system to perform the *k*_*tr*_ measurements. T-clipped skinned papillary fibers were cycled through minimal (pCa 9.0), submaximal (pCa 5.7), and maximal (pCa 4.5) calcium solutions, and between each pCa solution, the fibers were allowed to reach steady-state force before moving to the next pCa solution. The *k*_tr_ was assessed by allowing papillary fiber to reach steady-state force in pCa 5.7, before imposing a 20% length-shortening over 20 ms before a rapid (∼1 ms) stretch back to its original length. The *k*_tr_ was calculated by fitting the force redeveloped in the fiber, after this slack-stretch treatment against the time required to one-phase association curve. For the effect of S2 proteins on the *k*_tr,_ the fibers were permeabilized with hS2 proteins for 300 s in pCa 9.0 and later exposed to pCa 5.7 and pCa 4.5 solutions that also contained S2 proteins.

### PKA and λ-phosphatase treatment of skinned papillary fibers

The PKA solution was prepared by adding DTT to a final concentration of 6 mM to 800 μl of pCa 9.0 or a relaxing solution that was further added to 400 Unit PKA (Cat. # P2645, Sigma) to yield 0.5 U PKA/μl. Skinned papillary fibers were incubated in this PKA solution at RT for 90 min. Lambda phosphatase (Cat. # P0753S, New England Biolabs) was diluted 100-fold in pCa 9.0 solution to give final yield of 4U phosphatase/μl. Skinned fibers were treated with this phosphatase solution for 90 min at RT. The effect of PKA and phosphatase on the levels of phosphorylation in cMyBP-C was estimated by running a Western blot with cMyBP-C phospho-specific antibody (p273, p282, and p302) and an antibody to C0 domain of cMyBP-C. Antibody against β-actin (Cat. # sc-47778, Santa Cruz Biotechnology) was used as the loading control (Fig. S5, Table S2).

### Statistical analysis

The experimental data were analyzed in Microsoft Excel and GraphPad Prism 7. All the data are represented as the mean ± SEM. Experiments and data analyses were performed in a blinded manner wherever possible. Both male and female mice were included in the study. All data were analyzed with statistical significance calculated by performing ordinary one-way ANOVA with Tukey’s multiple comparison test with single pooled variance with significance accepted at *p* < 0.05. Degrees of freedom for the numerator and denominator along with F and *p* values for one-way ANOVA performed for each figure are outlined in Table 7. Each figure legend in the article has included a respective statistical analysis.

**Table 7:** One-way ANOVA analysis with main factors and interactions

Figure	F(DFn, DFd)	*p*
Figure 2*D*	F (7, 16) = 14.32	*p* < 0.0001
Figure 2*E*	F (7, 16) = 56.88	*p* < 0.0001
Figure 3*A*	F (5, 12) = 11.74	*p* = 0.0003
Figure 3*B*	F (5, 12) = 3.771	*p* = 0.0277
Figure 3*C*	F (5, 12) = 8.685	*p* = 0.0011
Figure 3*D*	F (5, 12) = 4.353	*p* = 0.0172
Figure 3*E*	F (5, 12) = 9.801	*p* = 0.0006
Figure 3*F*	F (5, 12) = 8.221	*p* = 0.0014
Figure 4*E*	F (7, 16) = 399.1	*p* < 0.0001
Figure 4*F*	F (7, 16) = 99.61	*p* < 0.0001
Figure 5*C*	F (4, 20) = 38.62	*p* < 0.0001
Figure 5*E*	F (4, 20) = 74.56	*p* < 0.0001
Figure 5*F*	F (4, 20) = 73.27	*p* < 0.0001
Figure 6*B*	F (4, 20) = 16.24	*p* < 0.0001
Figure 6*D*	F (4, 20) = 23.93	*p* < 0.0001
Figure 7*B*	F (3, 8) = 2.254	*p* = 0.1593
Figure 7*D*	F (3, 8) = 419.3	*p* < 0.0001

## Data availability

All data that support the findings of this study are contained within the article and its supporting information.

## Supporting information

This article contains supporting information (9, 42).

## Conflict of interest

S. S. provides consulting and collaborative research studies to the Leducq Foundation (CURE-PLAN), Red Saree Inc, Greater Cincinnati Tamil Sangam, AstraZeneca, MyoKardia, Merck, and Amgen, but such work is unrelated to the content of this article. R. R. S. has been a postdoctoral fellow of Amgen, starting from June 2019, and performs research at the University of Cincinnati. J. W. M. declares no conflicts of interest with the contents of this article.
