# Supplementary material for: Mutations in myosin S2 alter cardiac myosin-binding protein-C interaction in hypertrophic cardiomyopathy in a phosphorylation-dependent manner
Source: J Biol Chem. 2021 May 27;297(1):100836. doi: 10.1016/j.jbc.2021.100836 (PMC8239744; doi:10.1016/j.jbc.2021.100836)
Supplement: Supplemental Figures S1–S12 and Tables S1–S3 [file mmc1.pdf]

Supporting Data Information

Mutations in myosin S2 alter cardiac myosin binding protein-C interaction in hypertrophic cardiomyopathy in a phosphorylation-dependent manner

Rohit R. Singh[1], James W. McNamara[1] and Sakthivel Sadayappan[1*]

[1]From the Heart, Lung and Vascular Institute, Department of Internal Medicine, Division of Cardiovascular Health and Disease, University of Cincinnati, Cincinnati, OH 45236

Running title: Interaction of Myosin S2 and cMyBP-C in HCM

*To whom correspondence should be addressed: Sakthivel Sadayappan, PhD, MBA, Heart, Lung and Vascular Institute, Department of Internal Medicine, University of Cincinnati, 231 Albert Sabin Way, Cincinnati, OH 45267, USA. Phone: +15135587498. Email: sadayasl@ucmail.uc.edu

Supplemental Figures

```
O70468|Mybpc3_MOUSE      MPEPGKKPVSAFNKKPRSAEVTAGSAAVFEAETERSGVMVRWQRDGSDITANDKYGLAAE      60
Q14896|MYBPC3_HUMAN      MPEPGKKPVSAFSKKPRSVEVAAGSPAVFEAETERAGVKVRWQRGGSDISASNKYGLATE      60
                         ************.*****.**:*** *********:** *****.****:*.:*****:*

O70468|Mybpc3_MOUSE      GKRHTLTVRDASPDDQGSYAVIAGSSKVKFDLKVTEPAPPEK-----AESEVAPGAPEEV      115
Q14896|MYBPC3_HUMAN      GTRHTLTVREVGPADQGSYAVIAGSSKVKFDLKVIEAEKAEPMLAPAPAPAEATGAPGEA      120
                         *.*******:..* ******************** *    *           * *** *.

O70468|Mybpc3_MOUSE      PAPATELEESVSSPEGSVSVTQDGSAAEHQGAPDDPIGLFLMRPQDGEVTVGGSIVFSAR      175
Q14896|MYBPC3_HUMAN      PAPAAELGESAPSPKGSSSAALNG---PTPGAPDDPIGLFVMRPQDGEVTVGGSITFSAR      177
                         ****:** **. **:** *.: :*      **********:**************.****

O70468|Mybpc3_MOUSE      VAGASLLKPPVVKWFKGKWVDLSSKVGQHLQLHDSYDRASKVYLFELHITDAQTTSAGGY      235
Q14896|MYBPC3_HUMAN      VAGASLLKPPVVKWFKGKWVDLSSKVGQHLQLHDSYDRASKVYLFELHITDAQPAFTGSY      237
                         ***************************************************** : :*.*

O70468|Mybpc3_MOUSE      RCEVSTKDKFDSCNFNLTVHEAIGSGDLDLRSAFRRISLAGAGRRTSDSHEDAGTPDFSS      295
Q14896|MYBPC3_HUMAN      RCEVSTKDKFDCSNFNLTVHEAMGTGDLDLLSAFRRISLAGGGRRISDSHEDTGILDFSS      297
                         ***********..*********:*:***** **********.*** ******:*  ****

O70468|Mybpc3_MOUSE      LLKKRDSFR--RDSKLEAPAEEDVWEILRQAPPSEYERIAFQHGVEACHRPLKRLKGMKQ      353
Q14896|MYBPC3_HUMAN      LLKKRDSFRTPRDSKLEAPAEEDVWEILRQAPPSEYERIAFQYGVTDLRGMLKRLKGMRR      357
                         *********  *******************************:**   :  *******::

O70468|Mybpc3_MOUSE      DEKKSTAFQKKLEPAYQVNKGHKIRLTVELADPDAEVKWLKNGQEIQMSGSKYIFESVGA      413
Q14896|MYBPC3_HUMAN      DEKKSTAFQKKLEPAYQVSKGHKIRLTVELADHDAEVKWLKNGQEIQMSGSKYIFESIGA      417
                         ******************.************* ************************:**

O70468|Mybpc3_MOUSE      KRTLTISQCSLADDAAYQCVVGGEKCSTELFVKEP 448
Q14896|MYBPC3_HUMAN      KRTLTISQCSLADDAAYQCVVGGEKCSTELFVKEP 452
                         ***********************************
```

Figure S1: Amino acid sequence alignment (Clustal W) for C0-C2 domains of cMyBP-C between mouse (*Mybpc3*) and human (*MYBPC3*). The sequence alignment of the C0 (green), C1(yellow), M- (pink) and C2 domains (cyan). PKA phosphorylatable serines are in grey. Conserved amino acid residues are indicated by asterisk '*'; colon ':' represents conserved amino acids with strongly similar properties, and period '.' represents conserved residues with weakly similar properties. Percent Identity Matrix gave a score of 83.82% of identity between the C0-C2 domain of mouse and human cMyBP-C. O70468 and Q14896 are UniProt IDs for *MYBPC3* of mouse and human, respectively.

```
P13533|MYH6_HUMAN      PLLKSAETEKEMATMKEEFGRIKETLEKSEARRKELEEKMVSLLQEKNDLQLQVQAEQDN 60
P12883|MYH7_HUMAN      PLLKSAEREKEMASMKEEFTRLKEALEKSEARRKELEEKMVSLLQEKNDLQLQVQAEQDN 60
Q02566|MYH6_MOUSE      PLLKSAETEKEMANMKEEFGRVKDALEKSEARRKELEEKMVSLLQEKNDLQLQVQAEQDN 60
Q91Z83|MYH7_MOUSE      PLLKSAETEKEMATMKEEFGRVKDALEKSEARRKELEEKMVSLLQEKNDLQLQVQAEQDN 60
                       ******* *****.***** *:*::***********************************

P13533|MYH6_HUMAN      LNDAEERCDQLIKNKIQLEAKVKEMNERLEDEEEMNAELTAKKRKLEDECSELKKDIDDL 120
P12883|MYH7_HUMAN      LADAEERCDQLIKNKIQLEAKVKEMNERLEDEEEMNAELTAKKRKLEDECSELKRDIDDL 120
Q02566|MYH6_MOUSE      LNDAEERCDQLIKNKIQLEAKVKEMTERLEDEEEMNAELTAKKRKLEDECSELKKDIDDL 120
Q91Z83|MYH7_MOUSE      LADAEERCDQLIKNKIQLEAKVKEMTERLEDEEEMNAELTAKKRKLEDECSELKRDIDDL 120
                       * ***********************.****************************:*****

P13533|MYH6_HUMAN      ELTLAK  126
P12883|MYH7_HUMAN      ELTLAK  126
Q02566|MYH6_MOUSE      ELTLAK  126
Q91Z83|MYH7_MOUSE      ELTLAK  126
                       ******

Percent Identity Matrix
P13533|MYH6_HUMAN  100.00   94.44   96.03   95.24
P12883|MYH7_HUMAN   94.44  100.00   93.65   95.24
Q02566|MYH6_MOUSE   96.03   93.65  100.00   97.62
Q91Z83|MYH7_MOUSE   95.24   95.24   97.62  100.00
```

Figure S2: Proximal myosin S2 126 amino acid sequence alignment (Clustal W) for α-myosin heavy chain (*MYH6*) and β-myosin heavy chain (*MYH7*) between mouse and human. Highlighted residues are indicative of conserved amino acids for HCM mutations (R870H (Yellow), E924K (green) and E930Δ (cyan)) in human *MYH7*. Conserved amino acid residues are indicated by asterisk '*'; colon ':' represents conserved amino acids with strongly similar properties, and period '.' represents conserved residues with weakly similar properties. Percent Identity Matrix gave a score of greater than 92% identity between proximal myosin S2 of mouse and human α and β myosin heavy chain. P13533 and P12883 are UniProt IDs of *MYH6* and *MYH7* for human, respectively. Q02566 and Q91Z83 are Uniprot IDs of *MYH6* and *MYH7* for mouse, respectively.

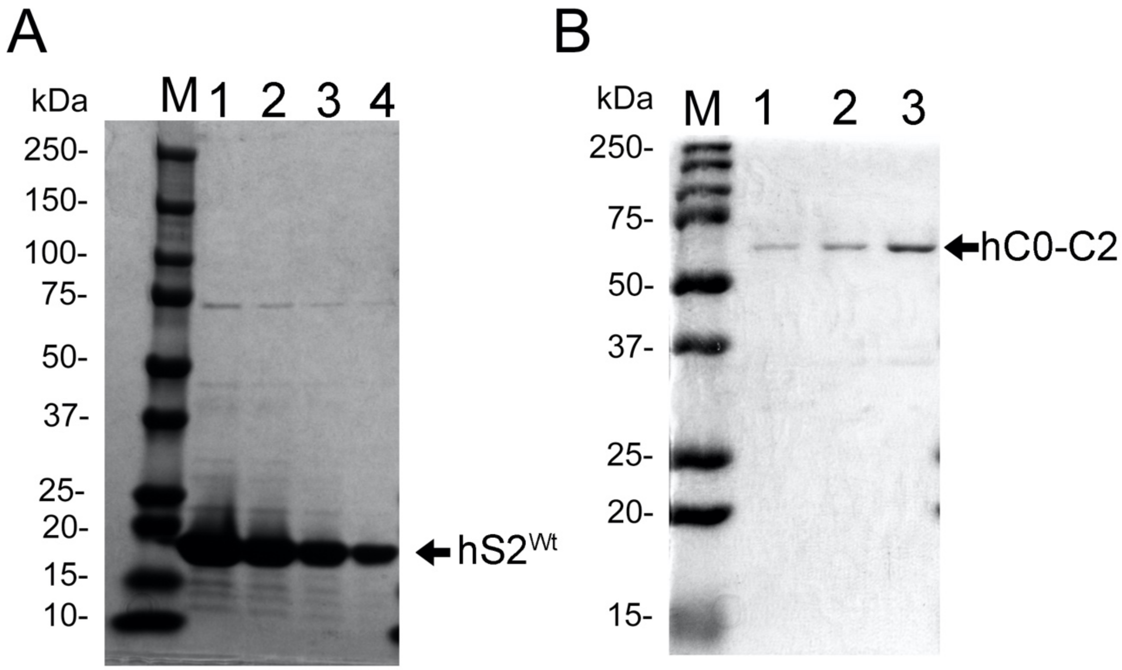

Figure S3. Representative images of Coomassie-stained SDS-PAGE gel showing the purified recombinant hS2^{Wt} and hC0-C2 proteins. Various concentrations of NiNTA-purified recombinant (**A**) hS2^{Wt} (Lane 1, 7.5µg; Lane 2, 5.0µg; Lane 3, 2.5µg and Lane 4, 1.0 µg) and (**B**) hC0-C2 proteins (Lane 1, 100ng; Lane 2, 200ng and Lane 3, 300ng) were loaded onto 4-15% gradient SDS-PAGE and stained with Coomassie blue for imaging. Precision Plus Protein Dual Color Standards (BioRad Catalog # 1610374) were used for standard molecular weight markers.

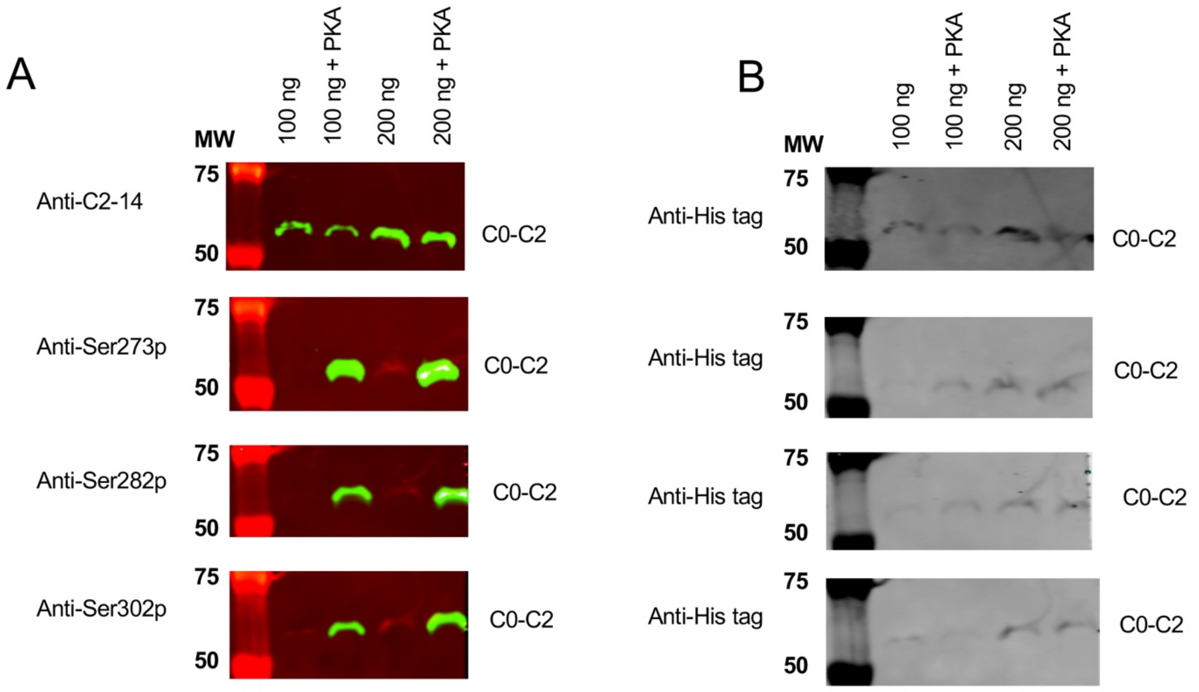

Figure S4. Phosphorylation of recombinant hC0-C2 proteins by PKA treatment. To determine the level of C0-C2 phosphorylation after PKA treatment, Western blot analyses were performed using phosphor-specific antibodies against Ser 273, Ser-282 and Ser-302 sites. (**A**) Representative Western blot analysis of recombinant hC0-C2 proteins using rabbit anti-phosphorylation Ser-273 antibody (anti-Ser273p), anti-phosphorylation Ser-282 antibody (anti-Ser-282p) and anti-phosphorylation Ser-302 antibody (anti-Ser-302p). Total protein levels were determined with rabbit anti-C2-14 (amino acids 2-14 of the C0 domain is the epitope). (**B**) Respective Western blots were further used to determine the total level of hC0-C2 proteins by using mouse anti-His-tag antibodies as the N'-region of hC0-C2 was tagged with His-tag. IR dye 680CD anti-mouse (red) and IR dye 800 CW anti-rabbit (green) were used as reporter or secondary antibody. Results confirmed that C0-C2 proteins were phosphorylated by PKA, compared to untreated controls.

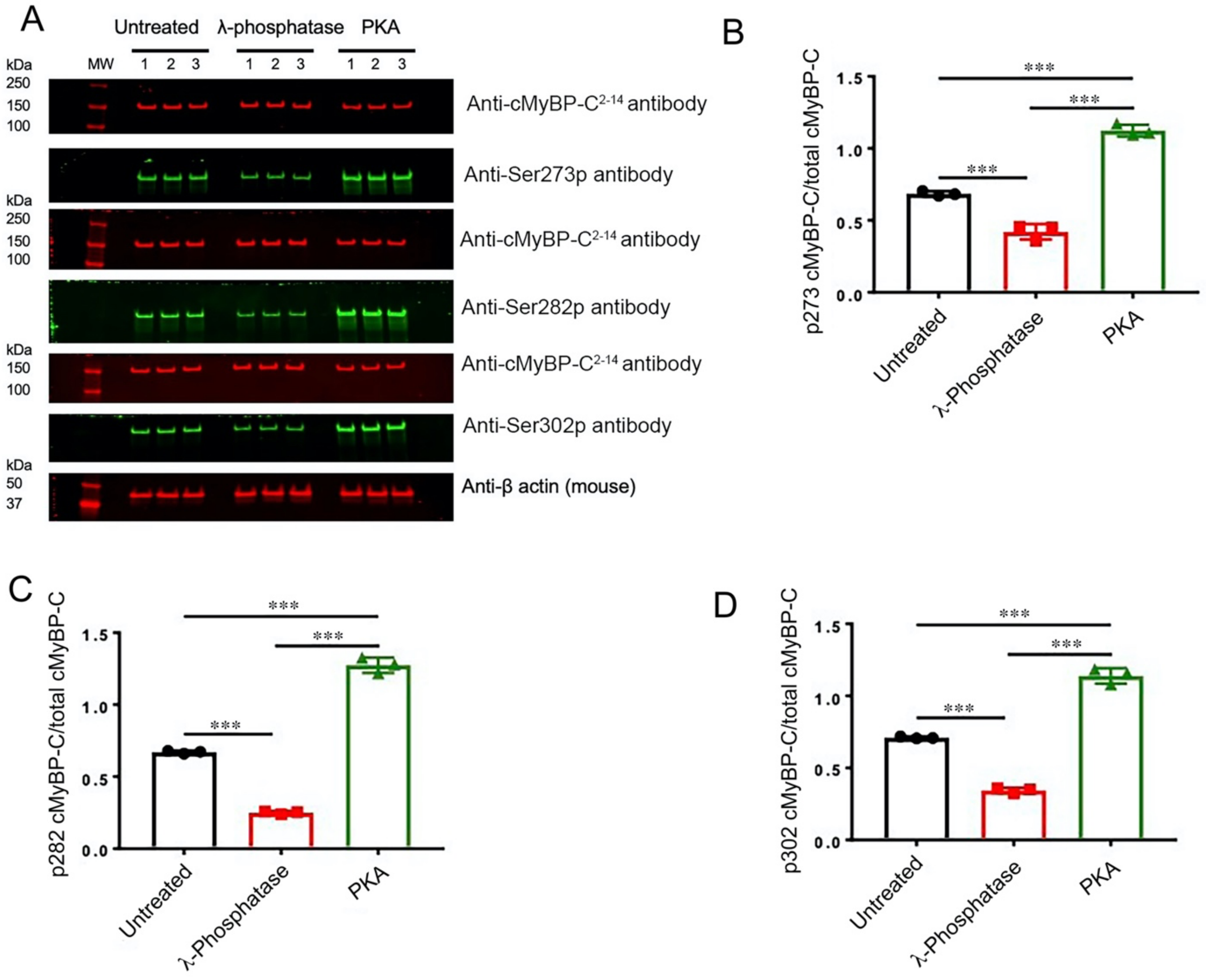

Figure S5. Phosphorylation of endogenous cMyBP-C protein in skinned papillary fibers by PKA and λ-phosphatase treatment. (**A**) To determine whether endogenous cMyBP-C in fibers was phosphorylated, Western blots were performed using protein samples from untreated fibers and fibers treated with either PKA or λ-phosphatase and blotted against either mouse anti-C0 domain and rabbit anti-phosphorylation Ser-273 (anti-Ser273p) antibody or rabbit anti-phosphorylation Ser-282 (anti-Ser282p) antibody or rabbit anti-phosphorylation Ser-302 (anti-Ser302p) antibody. Fibers from three different mice (animal ID: 1, ID: 2 and ID: 3) were used for treatment and loaded on same gel. Mouse antibody against β-actin was used as the loading control. IR dye 680CD anti-mouse (red) and IR dye 800 CW anti-rabbit (green) were used as reporter or secondary antibody. Phosphorylation status of each treatment was calculated by taking the ratio of intensity of bands for Ser-273 (**B**), Ser-282 (**C**) and Ser-302 (D) phosphorylation over intensity of the whole cMyBP-C band (top to bottom). Intensity was measured using Image Studio 4. Statistically significant value calculated with one-way ANOVA and Tukey's pooled multiple variance test. ± values are S.E.M. for reported values. Refer to Table S2 for analysis of main factors and interactions. (n=3) ****P* <0.001 for mean intensity compared against each group.

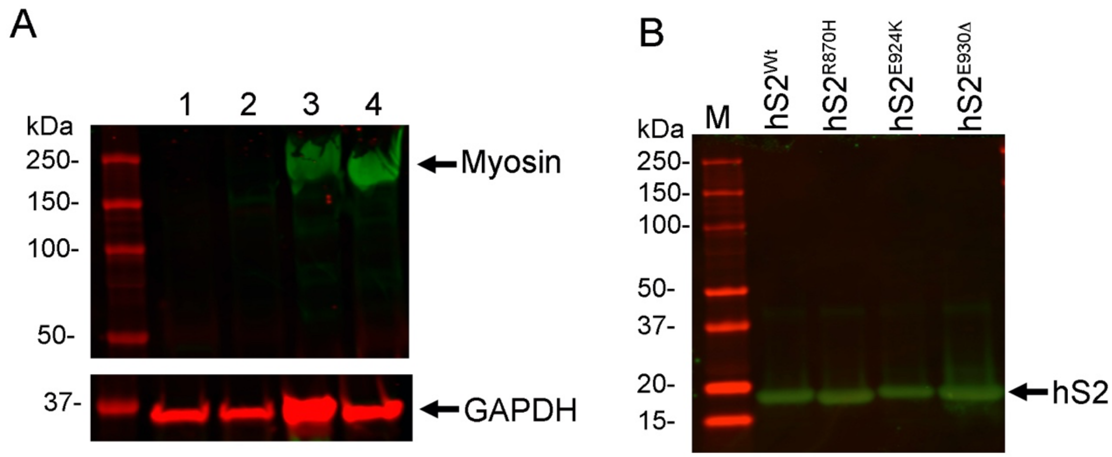

Figure S6. Validation of newly generated rabbit anti-human S2 antibodies by Western blot analyses. (**A**) Whole tissue lysates of liver (Lane 1), small intestine (Lane 2), skeletal gastrocnemius muscle (Lane 3) and heart (Lane 4) were analyzed with the newly generated rabbit anti-human S2 antibody (ProSci Inc, Poway, CA) and glyceraldehyde 3-phosphate dehydrogenase (GAPDH) with mouse anti-GAPDH monoclonal antibody (Fitzgerald Industries International, 10R-G109a). (**B**) Purified human S2 proteins with their mutant variants, as detected by novel rabbit anti-human S2 antibody. Secondary antibody of IR dye 680CD anti-mouse (red) and IR dye 800 CW anti-rabbit (green) were used. Precision Plus Protein™ Kaleidoscope™ Prestained Protein Standards (BioRad, Cat #1610375) were used for standard molecular weight markers.

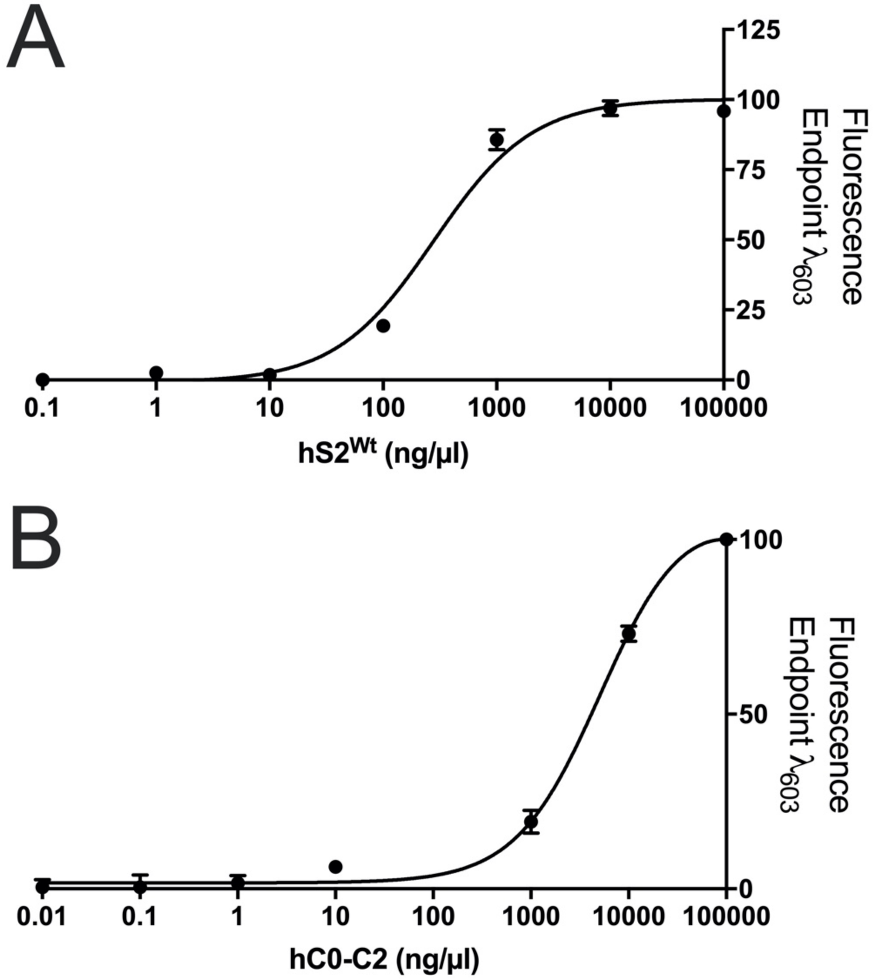

Figure S7. Detection limits for hS2 and hC0-C2 recombinant proteins by the anti-rabbit S2-specific antibody and C0-C2-specific antibody for SPBA. (**A**) hS2 protein was detected from the range of 0 to 6000 µM by primary 1:250 diluted hS2 rabbit antibody, followed by secondary goat anti-rabbit Alexa 563 antibody. (**B**) hC0-C2 protein was detected from the range of 0 to 2000 µM by primary 1:250 diluted hC0-C2 rabbit antibody, followed by secondary goat anti-rabbit Alexa 563 antibody. The x-axis is expressed in $\log_{10}$ scale to highlight the detectable range of myosin S2 (n = 4, fitted to one site: total binding curve by GraphPad Prism).

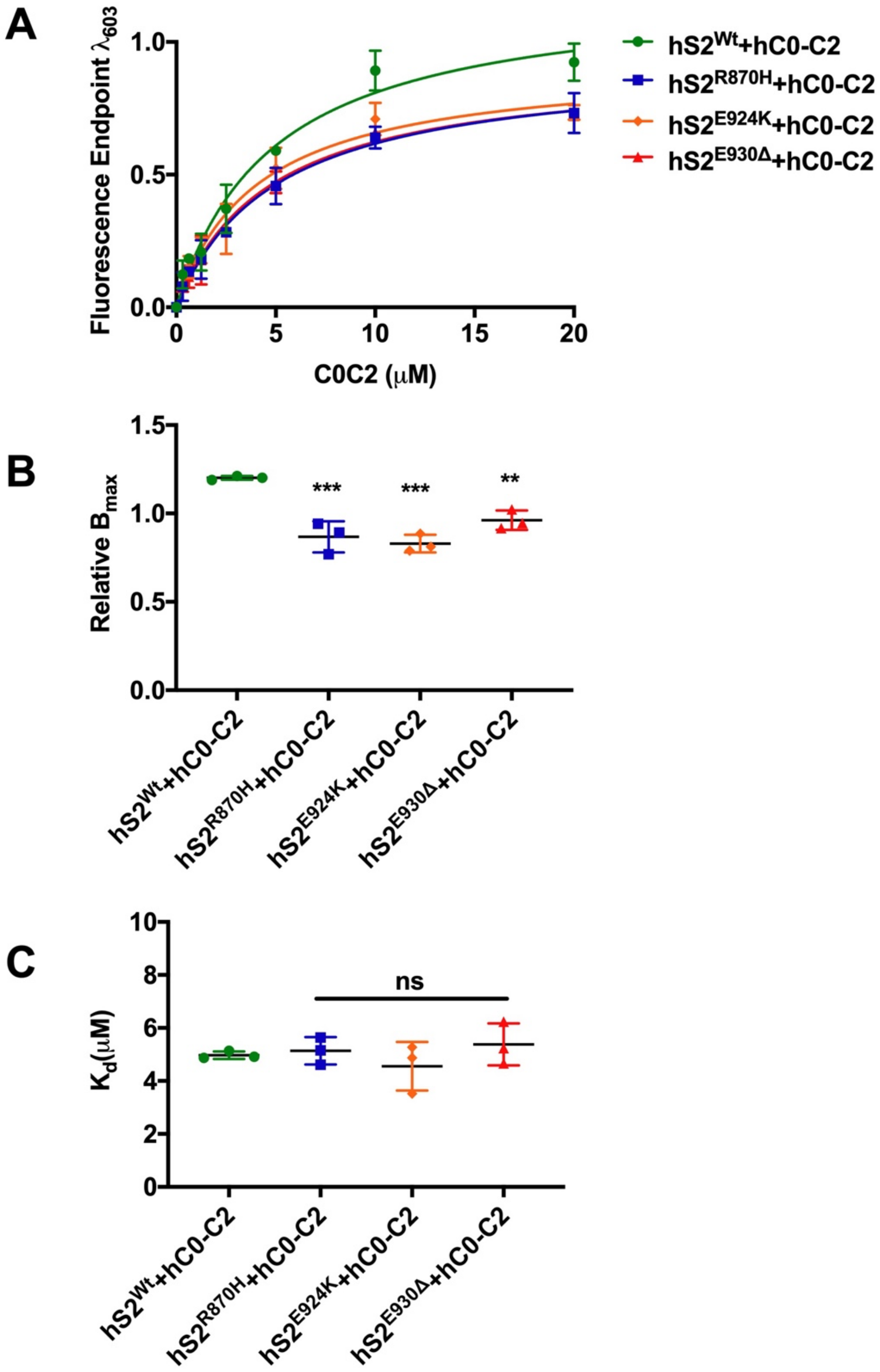

Figure S8. Binding of hS2 to hC0-C2 proteins. S2 protein was coated onto the plate and detected by antibody against hC0-C2 protein using SPBA. (**A**) Binding curve for $hS2^{Wt}$ (green), $hS2^{R870H}$ (blue), $hS2^{E924K}$ (orange) and $hS2^{E930\Delta}$ (red) to hC0-C2 proteins. (**B**) Significant decrease in relative B_{max} for hS2 mutants. (**C**) Non-significant change in dissociation constant (K_d) for hS2 mutants to hC0-C2 proteins. One-way ANOVA with Tukey's multiple comparison test was performed with single pooled variance. n=3 with triplicates for each hS2 protein. Refer to Table S2 for the one-way ANOVA analysis with main factors and interactions. *P <0.05, **P <0.01, and ***P <0.001 versus $hS2^{Wt/}$hC0-C2 proteins

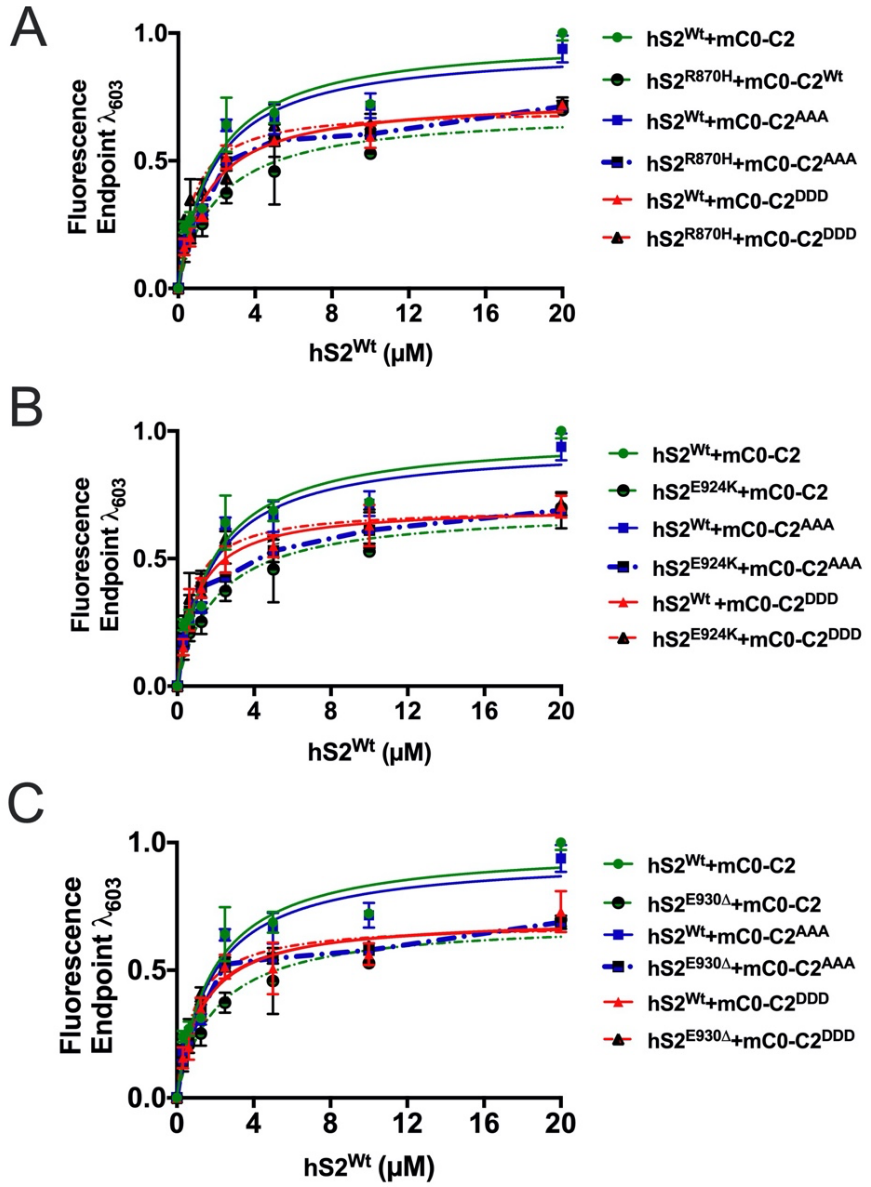

Figure S9. Binding of hS2 to mC0-C2. mC0-C2 was coated onto the microtiter plate, and binding was detected by antibody against hS2 protein by SPBA. Binding curve of hS2^{Wt} (solid), (**A**) hS2^{R870H} (dotted), (**B**) hS2^{E924K} (dotted) and (**C**) hS2$^{E930\Delta}$ (dotted) to mC0-C2^{Wt} (green), mC0-C2^{AAA} (blue) and mC0-C2^{DDD}. n=3 with triplicates for each hS2 protein. One-way ANOVA with Tukey's multiple comparison test was performed with single pooled variance. Refer to Table 2 and 7 of main text for comparison of B_{max} and K_d values across all binding interactions between hS2 and mC0-C2.

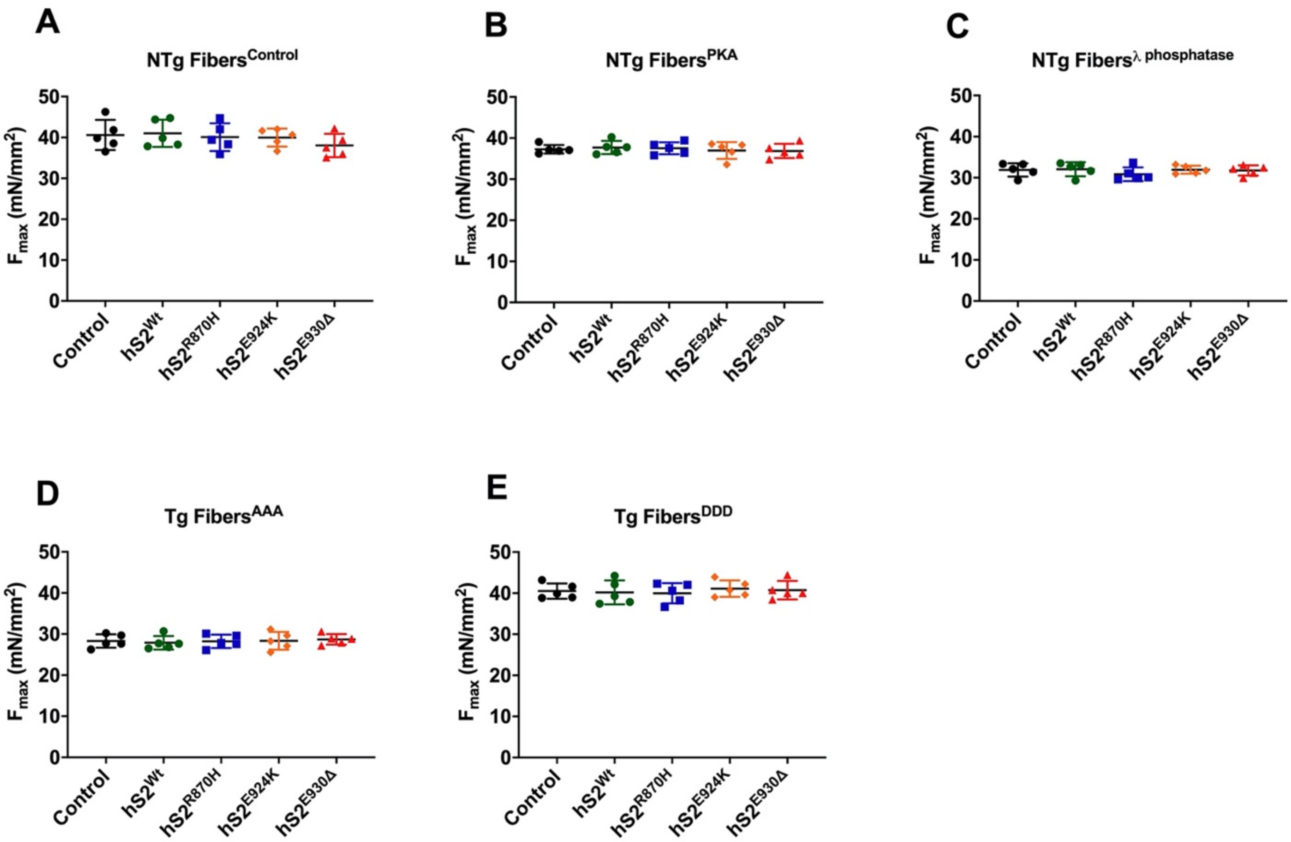

Figure S10. Maximal force (F_{max}) produced per cross section area of fibers treated with 9 µM S2 proteins at pCa 4.5. Non-significant change in force produced in fibers of (**A**) NTg fibers^{control}, (**B**) NTg fibers^{PKA}, (**C**) NTg fibers$^{\lambda\text{-phosphatase}}$, (**D**) Tg Fibers^{AAA} and (**E**) Tg Fibers^{DDD} in the presence of 9 µM of hS2^{Wt} (green), hS2^{R870H} (blue), hS2^{E924K} (orange) and hS2$^{E930\Delta}$ (red). One-way ANOVA with Tukey's multiple comparison test was performed with single pooled variance. n=5, with one fiber per animal. No significance was determined among the groups. Refer to Table S2 for one-way ANOVA analysis with main factors and interactions.

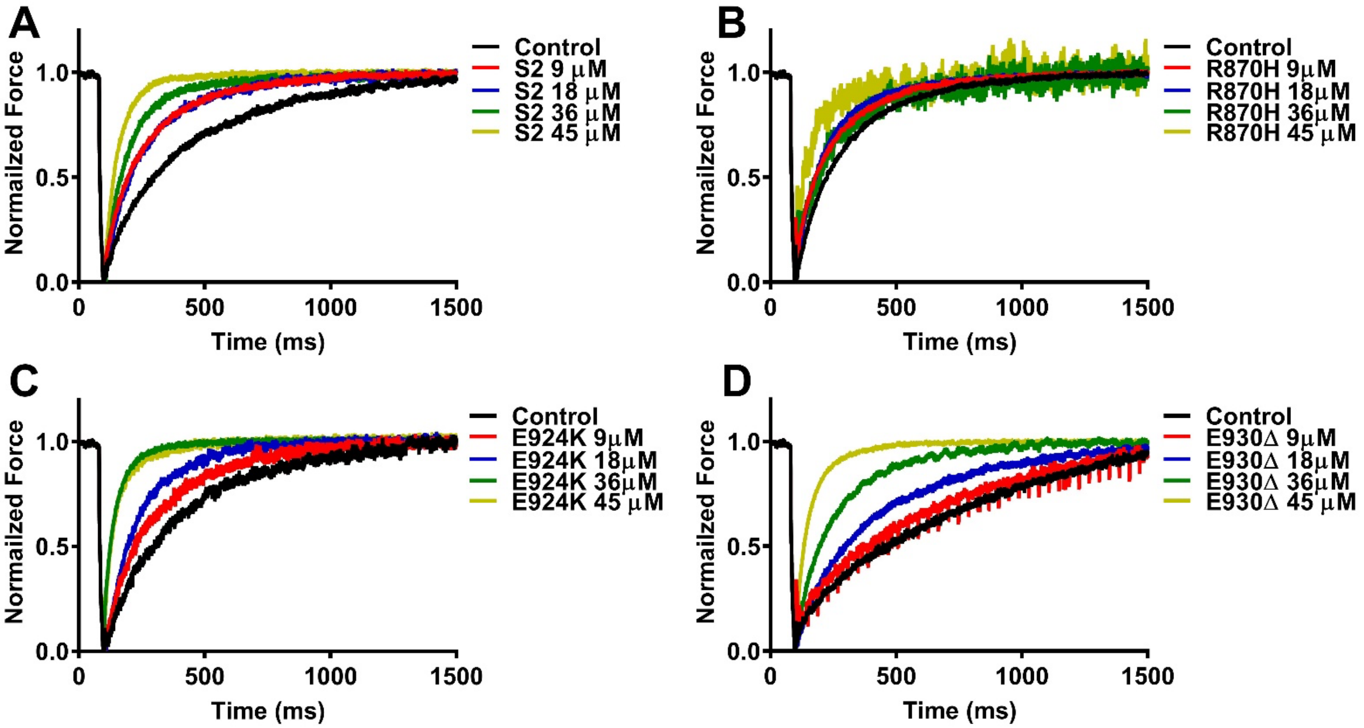

Figure S11. Dose-dependent effect of S2 proteins over the rate of force redevelopment. (**A-D**) k_{tr} raw traces for fibers that were permeabilized with (9-45µM) $S2^{Wt}$, $S2^{R870H}$, $S2^{E924K}$ and $S2^{E930\Delta}$, with untreated control (black), 9 µM (red), 18 µM (blue), 36 µM (green) and 45 µM (yellow) for each of the S2 proteins.

A

```
          838 PLLKSAE REKEMAS
852 MKEEFTR LKEALEK SEARRKE LEEKMVS
880 LLQEKND LQLQVQA EQDNLAD AEERCDQ
908 LIKNKIQ LEAKVKE MNERLED EEEMNAE
936 LTAKKRK LEDECSE LKRDIDD LELTLAK
```

B

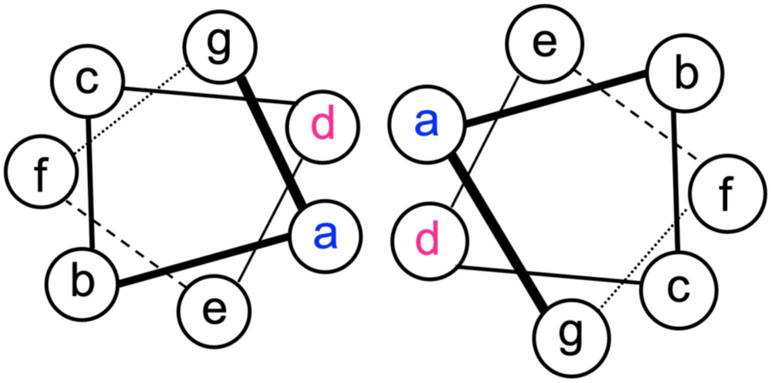

Figure S12. The heptad repeats of the myosin S2 showing the locations of the three mutations. **A**, The amino acid sequence of myosin S2 labeled with position a and d which are occupied by hydrophobic residues. The amino acids in position a and d form the hydrophobic residues to stabilize the coiled-coil structure of two α- helices S2 region. **B**, The 7-fold repeat of both strands is shown. HCM mutations (R870H (Yellow), E924K (green) and E930Δ (cyan)) in *MYH7* of human affect positions a, c and d, respectively.

Supplementary Tables

Table S1. Gene variants in the proximal region of proximal S2

#	Proximal S2 mutations	Charge Change
1	S842G	Polar Uncharged → Non-polar
2	E846Q	Negative → Polar Uncharged
3	K847Del	Positive → Deletion
4	K847E	Positive → Negative
5	E848G	Negative → Non-polar
6	M852T	Non-Polar → Polar Uncharged
7	R858H	Positive → Positve
8	R858S	Positive → Polar Uncharged
9	R858P	Positive → Non-polar
10	R858C	Positive → Non-polar
11	K865R	Positive → Positive
12	R869H	Positive → Positive
13	R869G	Positive → Non-polar
14	R870C	Positive → Non-polar
15	R870H	Positive → Positive
16	E875Del	Negative → Deletion
17	M877K	Non-polar → Positive
18	M877I	Non-polar → Non-polar
19	V878A	Non-polar → Non-polar
20	Q882E	Polar Uncharged → Negative
21	E883Del	Negative → Deletion
22	N885K	Polar Uncharged → Positive
23	E894G	Negative → Non-polar
24	A901G	Non-polar → Non-polar
25	C905F	Non-polar → Non-polar,Aromatic
26	D906G	Negative → Non-polar
27	L908V	Non-polar → Non-polar
28	L908Q	Non-polar → Polar Uncharged
29	I909M	Non-polar → Non-polar
30	E921K	Negative → Positive
31	E924K	Negative → Positive
32	E924Q	Negative → Polar Uncharged
33	E927K	Negative → Positive
34	E927Del	Negative → Deletion
35	D928V	Negative → Non-polar
36	D928N	Negative → Polar Uncharged
37	E930K	Negative → Positive
38	E930Del	Negative → Deletion
39	E930Q	Negative → Polar Uncharged
40	E931K	Negative → Positive
41	E949K	Negative → Positive
42	D953H	Negative → Positive

Charge reversal	8
Polar to Non-polar	9
Deletion of charged residues	5
Non-polar to Polar Uncharged	3
Polar charged side chain to Polar uncharged side chain	7
No change in charge of the residue	9
Non-polar to Non-polar Aromatic side chain	1
**Total**	**42**

The listed mutations are localized in the proximal region of S2 and cause hypertrophic cardiomyopathy. Various color codes represent the charge of the mutant amino acids. As indicated, mutations in the proximal region of S2 are predominantly positively charged. In the present study, we have used R870H, E924K and E930Δ (Del) mutations to understand the molecular consequences *in vitro* (1, 2).
https://www.uniprot.org/uniprot/P12883, UniProt Id = P12883 (Human, *MYH7*)

Table S2. One-way ANOVA analysis with main factors and interactions

Suppl Figure; Panel	F(DFn, DFd)	*P*
S5 B (ser-273p)	F (2, 6) = 231.5	P<0.0001
S5 B (ser-282p)	F (2, 6) = 784.6	P<0.0001
S5 B (ser-302p)	F (2, 6) = 436.9	P<0.0001
S7 B	F (3, 8) = 24.85	P=0.0002
S7 C	F (3, 8) = 0.8245	P=0.5163
S10 A	F (4, 20) = 0.6605	P=0.6266
S10 B	F (4, 20) = 0.233	P=0.9165
S10 C	F (4, 20) = 0.5405	P=0.7078
S10 D	F (4, 20) = 0.1506	P=0.9605
S10 E	F (4, 20) = 0.1862	P=0.9429

Table S3: Myosin S2 cDNA primers utilized to generate recombinant hS2 proteins with mutations

Mutagenesis Primers	5' to 3' sequence
Myosin S2_R870H_F	GAGGCTCGCCACAAGGAGCTG
Myosin S2_R870H_R	GGACTTCTCTAGCGCCTC
Myosin S2_E924K_F	GGAGATGAACAAGAGGCTGGAGG
Myosin S2_E924K_R	TTCACCTTGGCCTCCAGC
Myosin S2_E930Δ_F	GAGATGAATGCTGAGCTC
Myosin S2_E930Δ_R	CTCATCCTCCAGCCTCTC

DNA sequences were obtained from the UniProt ID:P12883 database.

References:

1. Homburger, J. R., Green, E. M., Caleshu, C., Sunitha, M. S., Taylor, R. E., Ruppel, K. M., Metpally, R. P., Colan, S. D., Michels, M., Day, S. M., Olivotto, I., Bustamante, C. D., Dewey, F. E., Ho, C. Y., Spudich, J. A., and Ashley, E. A. (2016) Multidimensional structure-function relationships in human beta-cardiac myosin from population-scale genetic variation. *Proc Natl Acad Sci U S A* **113**, 6701-6706

2. Trivedi, D. V., Adhikari, A. S., Sarkar, S. S., Ruppel, K. M., and Spudich, J. A. (2018) Hypertrophic cardiomyopathy and the myosin mesa: viewing an old disease in a new light. *Biophys Rev* **10**, 27-48
